# High Throughput Sequencing Analysis of the Immunoglobulin Heavy Chain Gene from Flow-Sorted B Cell Sub-Populations Define the Dynamics of Follicular Lymphoma Clonal Evolution

**DOI:** 10.1371/journal.pone.0134833

**Published:** 2015-09-01

**Authors:** Emanuela Carlotti, David Wrench, Guglielmo Rosignoli, Jacek Marzec, Ajanthah Sangaralingam, Lena Hazanov, Miri Michaeli, Simon Hallam, Tracy Chaplin, Sameena Iqbal, Maria Calaminici, Bryan Young, Ramit Mehr, Peter Campbell, Jude Fitzgibbon, John G. Gribben

**Affiliations:** 1 Centre for Haemato-Oncology, Barts Cancer Institute - a CR-UK Centre Of Excellence, Queen Mary University of London, London, United Kingdom; 2 Flow Cytometry Core Facility, Barts Cancer Institute - a CR-UK Centre Of Excellence, Queen Mary University of London, London, United Kingdom; 3 Centre for Molecular Oncology, Barts Cancer Institute - a CR-UK Centre Of Excellence, Queen Mary University of London, London, United Kingdom; 4 The Mina & Everard Goodman Faculty of Life Sciences, Bar-Ilan University, Ramat-Gan, Israel; 5 Cancer Genome Project, Wellcome Trust Sanger Institute, Hinxton, United Kingdom; University of Navarra, Center for Applied Medical Research, SPAIN

## Abstract

Understanding the dynamics of evolution of Follicular Lymphoma (FL) clones during disease progression is important for monitoring and targeting this tumor effectively. Genetic profiling of serial FL biopsies and examples of FL transmission following bone marrow transplant suggest that this disease may evolve by divergent evolution from a common ancestor cell. However where this ancestor cell resides and how it evolves is still unclear. The analysis of the pattern of somatic hypermutation of the immunoglobulin gene (Ig) is traditionally used for tracking the physiological clonal evolution of B cells within the germinal center and allows to discriminate those cells that have just entered the germinal center and display features of ancestor cells from those B cells that keep re-circulating across different lymphoid organs. Here we investigated the pattern of somatic hypermutation of the heavy chain of the immunoglobulin gene (IgH-VH) in 4 flow-sorted B cells subpopulations belonging to different stages of differentiation, from sequential lymph node biopsies of cases displaying diverse patterns of evolution, using the GS-FLX Titanium sequencing platform. We observed an unexpectedly high level of clonality, with hundreds of distinct tumor subclones in the different subpopulations from the same sample, the majority detected at a frequency <10^−2^. By using a lineage trees analysis we observed in all our FL and t-FL cases that the oligoclonal FL population was trapped in a narrow intermediate stage of maturation that maintains the capacity to undergo SHM, but was unable to further differentiate. The presence of such a complex architecture highlights challenges currently encountered in finding a cure for this disease.

## Introduction

Follicular lymphoma (FL) is an indolent disease characterized by interspersed episodes of remission and relapse, associated with a decreased sensitivity to therapeutic agents [1]. About 30% of cases undergo histological transformation to a more aggressive lymphoma, most commonly diffuse large B cell lymphoma (t-FL), an event associated with poor outcome [2, 3].

It is well established that the t(14;18) translocation is the founder genetic aberration of this disease and it is observed in about 90% of patients at diagnosis. This rearrangement provides a survival advantage to the B cell clone but it is not sufficient to initiate lymphoma [4, 5]. More recently, next generation sequencing studies have identified early driver mutations in chromatin regulator genes alongside genes that regulate the interaction of the tumor with its microenvironment [6]. These observations suggest that the second hit responsible for switching a long living B cell into a lymphoma cell could reside either in an altered epigenetic program or in a deviated interaction with other populations [6–9].

Cancer progression is now viewed as a genetic process that follows the same patterns observed in evolutionary biology. Studies in other hematological malignancies, [10–13] demonstrated that these tumors are characterized by an intra-tumor genetic heterogeneity and within individual patients, multiple subclones can coexist and initiate the disease. By investigating the variable region of the immunoglobulin heavy chain gene (IgH-VH) [14, 15] and performing genome wide analysis [16, 17], our group and others have proposed the existence of a more immature common progenitor cell (CPC) shared by tumor clones that are detected at relapse and transformation. It appears that this cell (or pool of cells) is rare and, based on the somatic hypermutation (SHM) pattern of IgH-VH, has already experienced the germinal center (GC). Indeed two reports of donor-derived FL occurring after bone marrow transplantation, [14, 18] including a study from our group, independently showed that this particular cell is long lived, with donors and recipients developing clonally related FL several years after transplant (range 3–10 years). Nevertheless, there are currently no distinctive markers capable of specifically targeting this ancestor. Even if it is plausible that the CPC has retained some properties of healthy GC B cells (i.e. proliferation/differentiation) little is known about the biological features that allow this committed cell to persist for extended periods of time or its role in lymphomagenesis.

In order to determine if we could detect this CPC and to gain insight into the dynamics of clonal evolution of FL tumor cells we investigated the SHM of IgH-VH using a high-throughput technology and DNA extracted from 4 flow-sorted subpopulations, corresponding to 4 different stages of B cell maturation, on sequential biopsies from 3 patients. Tumor infiltrating cells were detected in all the four subpopulations investigated and the level of clonality was far more complex than expected, with the majority of the clones present at frequencies below 10^−2^. Lineage tree analysis depicted a picture of a tumor cell population, entrapped in the GC with its SHM capacity intact. This complexity did not change when relapsed or FL transformed cases were compared. Irrespective of the huge number of different tumor related clones, in none of the sorted sub-populations were we able to identify a clone with a pattern of SHM compatible with that of the inferred CPC.

## Material and Methods

### Patient samples

**Fig 1:**
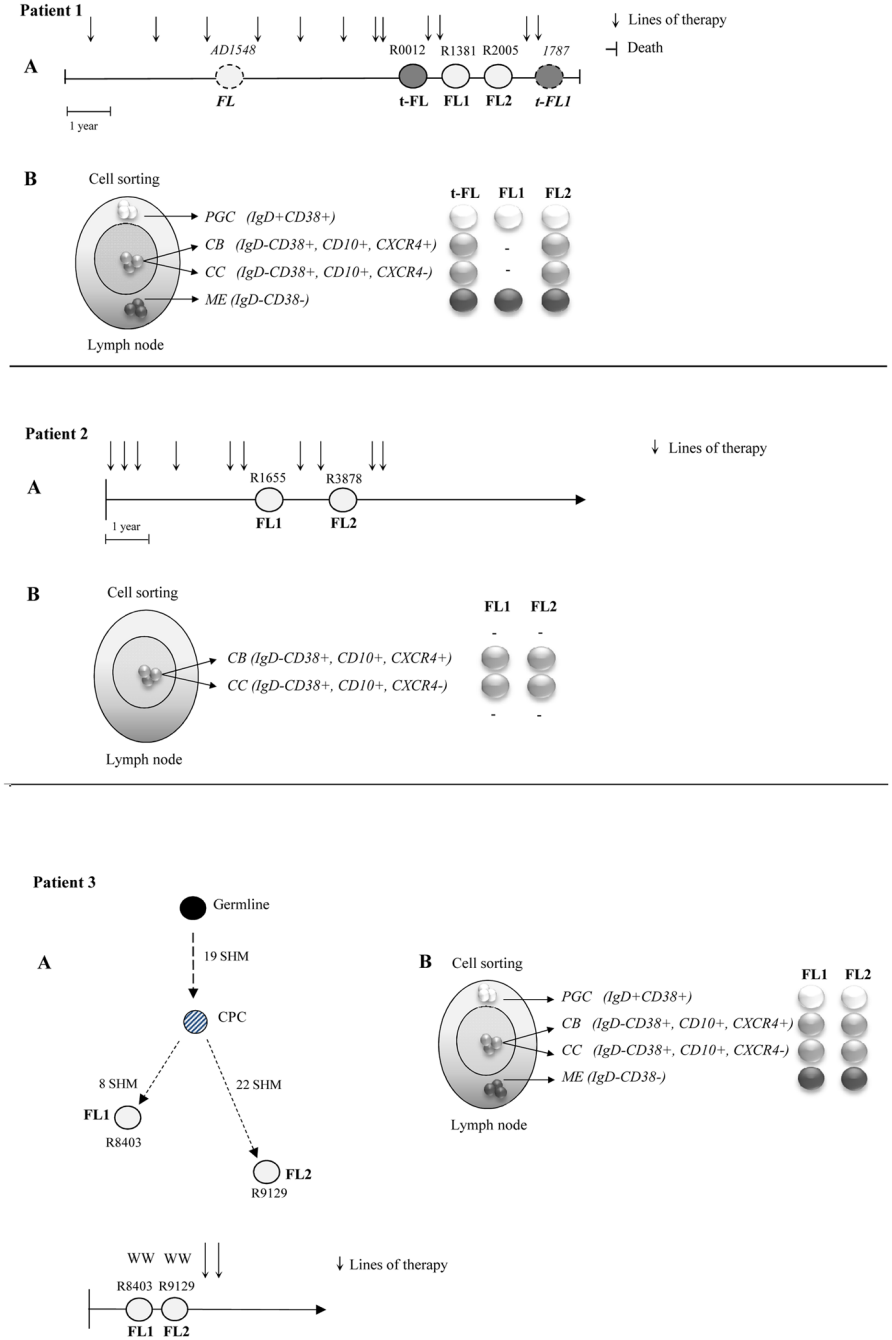
Characteristics of the patients and samples included in the study. (A) Three patients were included in the analysis: pt1 and pt2 displaying a pattern of direct evolution and pt3 of evolution through a CPC based on HH analysis. Biopsies were collected at different time points: at relapse/transformation (pt1 and pt2) and after a watch wait period (pt3). In pt 1 two additional biopsies (AD1548 and 1787) analyzed only on the SNP array are shown. In pt1 all samples had an identical MC whilst in pt2 the MC sequences from samples R1655 FL1 and R3878 FL2 showed 2 different dominant clones carrying 3 discriminating mutations (S7 Fig). Because these 3 bases also differ from the germline sequence a pattern of direct evolution could not be excluded. (B) Twenty-two samples, from 4 flow-sorted sub-populations, included in the deep sequencing study: pre-germinal center (PGC) (IgD+CD38+) white circles, the germinal center centroblasts (CB) (IgD+CD38+CD10+CXCR4+) and centrocytes, (CC) (IgD+CD38+CD10+CXCR4-) light grey circles and memory enriched (ME) (IgD-CD38-) dark grey circles. The 2 CC CD77- sorted sub-population from R0012 t-FL and R1381 FL1 biopsies, which were part of the deep sequencing study but not included in the final analysis are not shown. The FL sequences are depicted as white circles, the t-FL clones as grey circles, the germline sequence as black circles and the putative CPC sequence as a dashed grey circle. The 2 samples not included in the deep sequencing of IgH-VH gene are depicted as dotted circles. Each vertical arrow represents a line of therapy; the left hand vertical bar shows the time of diagnosis and the right hand vertical bar the time of death. FL = Follicular Lymphoma; t-FL = transformed Follicular Lymphoma; WW = watch and wait.

All biopsies were obtained after written informed consent in accordance with the Declaration of Helsinki and approval from the North East London Research Committee. Tumor samples were selected on the basis of the availability of viable frozen cells and the presence of a clonal rearrangement of IgH-VH detectable by homo/heteroduplex analysis (HH) [14, 19]. In total 9 biopsies from 3 patients were included in the study: two had a pattern compatible with direct evolution (Fig 1) and one with the existence of a CPC. Seven biopsies were included in the high-throughput analysis of IgH-VH and seven samples (of which five were overlapping) were studied using a SNP array strategy. All samples carried an IgH-VH3 rearranged major tumor clone (MC) (Table 1). Additional clinical information is provided in S1 Table.

**Table 1:** Clinical and molecular features of the 3 patients investigated by high-throughput sequencing.

Pt No	Sample ID±	Tumor	Site Biopsy	Histological grade	Sample type	Time from diagnosis years (months)	Pattern of evolution	t(14;18)	IgH-VH3 DH-JH Rearrangememnts	% homology IgH-VH	No SHM IgH-VH
1	*AD1548*	FL	Left axilla	Grade 1	P/R2	4 (6 mo)	Direct	MBR	IGH3-23*01/*03/*04/*05 DH3-2*01/JH4*02	86.69	36
	R0012	t-FL	Left axilla	DLBCL with small area FL grade	P	8 (3 mo)	Direct	MBR	IGH3-23*01/*03/*04/*05 DH3-2*01/JH4*02	86.69	36
	R1381	FL	Left axilla	N.A.	P	9 (4 mo)	Direct	MBR	IGH3-23*01/*03/*04/*05 DH3-2*01/JH4*02	86.69	36
	R2005	FL	Left axilla	Grade I	P	9 (9 mo)	Direct	MBR	IGH3-23*01/*03/*04/*05 DH3-2*01/JH4*02	86.69	36
	*1787*	t-FL	Left axilla	DLBCL	P	10 (9 mo)	Direct	MBR	IGH3-23*01/*03/*04/*05 DH3-2*01/JH4*02	86.69	36
2	R1655	FL	Left inguinal node	Grade 2	P	2 (8 mo)	Direct*	MBR-/mcr-	IGHV3-48*01 DH3-10*01/JH6*02	88.57	30
	R3878	FL	Left inguinal node	Grade 1	P	4	Direct*	MBR-/mcr-	IGHV3-48*01 DH3-10*01/JH6*02	88.57	30
3	R8403	FL	Right axilla	Grade 3A	SD	1 (4 mo)	Divergent/CPC	MBR	IGHV3-23*01/*04 DH4-23*04/JH6-02#	89.11	27
	R9129	FL	Right femoral node	Grade 1	SD	1 (9 mo)	Divergent/CPC	MBR	IGHV3-23*01/*04 DH3-3*01/JH5*01#	83.47	41

Three patients, 2 showing a pattern compatible with a direct evolution and 1 consistent with the existence of a CPC, all having a IgH-VH3 family clonal rearrangement were included in the study.

Samples AD1548 and 1787 from pt1, shown in italic, were not included in the high-throughput sequencing analysis of IgH-VH but only in the SNP CNV study.

^±^The sample ID corresponds to the cell suspension vial ID or DNA identification number.

P = progression, R = relapse, SD = stable disease; subsequent number relate to episode e.g. R2 = second relapse

N.A. = not available SHM = somatic hypermutation

FL = Follicular Lymphoma; t-FL = transformed Follicular Lymphoma

MBR stands for Major Breakpoint Region and mcr for minor cluster region

* Pt2 has 2 different mutations at the same base (a pattern compatible with direct evolution)

^#^ Samples R8403FL1 and R9129FL2 had 2 different DH/JH rearrangements but when aligned they showed the a similar CDR3 region, with additional 21 insertions in the sample R8403FL1 (see S6 Fig)

### Isolation and characterization of different B-cells subpopulations

**Fig 2:**
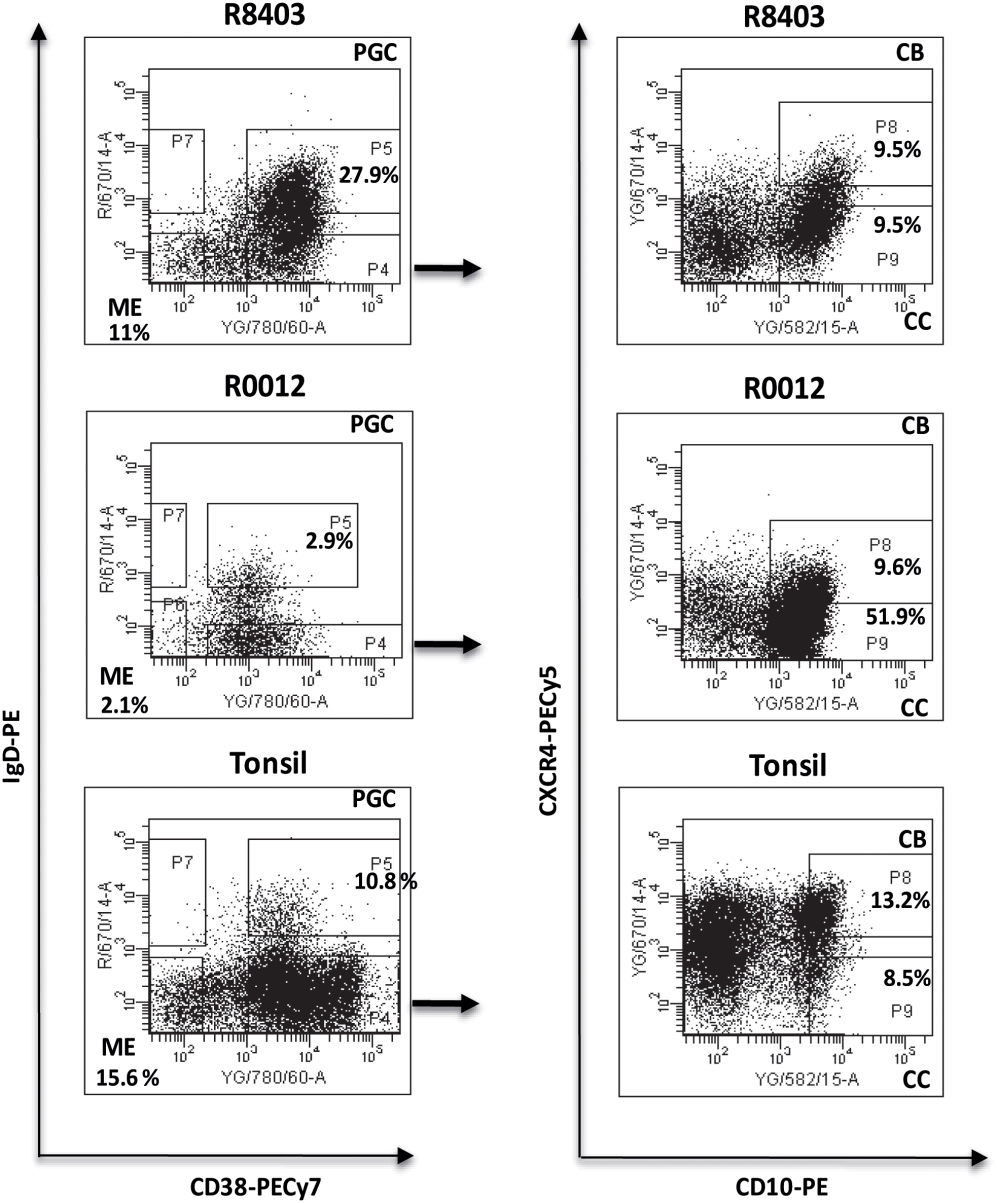
Flow-sorting of the 4 different B cell sub-populations. Lymph nodes cell suspensions from sequential biopsies obtained from patients with FL/t-FL were stained and sorted in 4 different populations according to the expression of IgD, CD38, CD10 and CXCR4. ME: IgD-CD38-; PGC: IgD+CD38+, CB: IgD-CD38+CD10+CXCR4+; CC: IgD-CD38+CD10+CXCR4-. Flow-cytometric identification of the 4 sub-populations in the sample R8403, patient 3 (top), sample R0012, patient 1 (middle) and tonsils (bottom). In the 2 patients samples the numbers indicate the percentage of the gated and sorted subsets whilst in the tonsil example the numbers are representative of 8 independent experiments on as many different biopsies.

Four different sub-populations were flow-sorted from reactive tonsils and FL biopsies cryopreserved cell suspensions and fluorescently labeled using the monoclonal antibodies: CD38-PE-Cy7 (clone HB7), CD10-PE (clone HI10a), CXCR4 PE-Cy5 (clone 12G5), IgD biotin (clone IA6-2), (Becton Dickinson; San Jose, CA, USA), CD77 FITC (clone 5B5) and anti-biotin APC (clone Bio3-18E7) (Miltenyi Biotec). Non-viable cells were excluded by simultaneously staining with DAPI (Sigma-Aldrich). Gates to distinguish the different subsets were set using cells suspensions from tonsils and the fluorescence minus one control. By flow sorting using a FACS Aria II (Becton Dickinson) fluorescently labeled cells were separated into four different subsets (Fig 2). Data acquisition and analysis was performed using DIVA software, version 6.13 (Becton Dickinson). The average purity in each sub-population was >90%. A further characterization of these populations was performed by staining with CD27 APC (clone M-T271) and CD20 FITC (clone 2H7) antibodies (Becton Dickinson; San Jose, CA, USA), cell cycle analysis, and Real-Time PCR analysis of *AICDA* and *mir155h6* expression (S1A–S1E Fig). Briefly, cell cycle analysis was carried out on frozen cell suspensions from tonsils, using the fixation and permeabilization buffers (eBioscience) and DAPI staining according to the commercial protocol (cells were re-suspended in 1.5mL of 2% FBS/PBS and 3μl of DAPI). RNA was extracted from tonsils and primary FL cell suspensions and the lymphoblastoid cell line NcNc, using the commercial kit RNAeasy Mini Kit (Qiagen). Standard cDNA synthesis was performed using the SuperscriptII Reverse Transcriptase and Random Primers (Invitrogen), according to the manufacturer's instructions and using 100 ng of RNA per reaction. Predesigned TaqMan Gene Expression assays for *AICDA*, *mir155h6* and *18S* genes were obtained from Applied Biosystem. *18S* was used as endogenous control. Both the target and the control genes were run in duplicate. The quantitative PCR was performed using the Taqman 7900HT Fast Real Time PCR System and the recommended manufacturer’s conditions. Gene expression values were then calculated using the ΔΔCt method.

### Preparation of the libraries and pyrosequencing

Genomic DNA was extracted with the DNeasy Blood and Tissue kit (Qiagen, UK). Thirty-eight libraries, from 32 different samples, (Fig 1) were prepared starting from 20–45 ng of DNA and using JH consensus and VH3-FR1 primers [19], modified with unique multiplex identifier (MID) tags (S2 Table). The PCR reaction was performed following a previously published protocol [14] with an amended annealing temperature of 61°C and 32 cycles. A control library was prepared with DNA from the A375 melanoma cell line and primers designed on the IgH germline locus. [20] In order to check for any bias introduced due to the different MID sequences used during the amplification, we generated 6 identical libraries performing independent PCR amplifications using a single set of primers and DNA extracted from the sample R8403NS and 2 different libraries using the sample R1381NS and 2 different primers (S2 Table). Thirty-eight amplicons (24 from flow-sorted sub-populations, 13 from NS samples and 1 control library) were purified and then pooled and sequenced using the Roche 454 Life Sciences Genome Sequencer FLX, following the manufacturer’s instructions for the Titanium series (454 Life Science, Roche).

### High-density SNP array

Five hundred ng of DNA, from sequential FL/t-FL biopsies (5 samples for pt1 and 2 for pt2) and from peripheral blood collected at the time of remission (pt1) were prepared and hybridized to Affymetrix Genome-Wide Human SNP Array Version 6.0 (Affimetrix, Santa Clara, CA, USA) according to the manufacturer's recommendations. Signal copy number variation (CNV) analysis was performed using the in house Genome Orientated Laboratory File (GOLF) platform. The IgH, IgL (κ and λ) loci (14q32, 2p11 and 22q11.2), the T cell receptor (TCRα, TCRβ, TCRδ and TCRγ) loci (14q11.2, 7q34 and 7p14) and sex chromosomes were excluded from the analysis. CNV for samples from pt2 (for which a germline sample was not available) was determined based on the log2 ratio of signal intensity from each lymphoma sample versus the pooled signal intensity of 16 unrelated control DNA samples.

### Data analysis

The sequential steps involved in the analysis of 454 reads are shown in S3 Fig. Tumor related reads (defined as the reads identical to the MC plus those sequences that based on the IgH-VH mutation pattern were clonally related) were identified according to the sequences detected by HH analysis. Sequences were separated according to each MID barcode and exported considering the first 8 bases of the sequence corresponding to the first 8 bases of the primer (i.e. the 4–5 bases of the barcode plus the 3–4 bases of the IGH primer) and a sequence length > 60 bases. Data generated from the A375 melanoma cell line were used to distinguish genuine SHM from artifacts according to a previously published algorithm [20]. Reads captured at least twice (either forward and reverse or only forward or reverse) that after manual examination of the pyrograms (Staden 2.0) showed a high quality trace were included in the study. Clones with SHM falling outside the IgH-VH region (DH-JH) (19% of total, range 0%- 65%, in R9129 ME and R0012 PGC respectively) and clones with a homology to the germline sequence higher than 90% (less than 1% of total) were excluded from the subsequent analysis. The IgH-VH reads were analyzed using IMGT/V-QUEST (http://www.imgt.org/). Sequences were aligned with ClustalW2 (http://www.ebi.ac.uk/Tools/msa/clustalw2/) and lineage trees generated using IgTree [21]. During the reconstruction we restricted alignment and analysis to the IgH-VH region. Amplicon sequences can be downloaded from the European Nucleotide Archive (ENA) using the accession number PRJEB9334.

## Results

### High-throughput sequencing analysis of flow-sorted B cells sub-populations

FL is a GC tumor and consists of an oligoclonal population. Taking advantage of this feature we decided to explore the complexity and to trace the clonal evolution of this disease over time by investigating the IgH-VH region in the whole tumor as well as in the selected sub-populations, using a high throughput sequencing strategy. We examined the IgH-VH repertoire in 7 lymph node biopsies, from three patients selected as showing patterns of direct evolution (pt1 and pt2) and CPC (pt3) (Fig 1A and 1B). In total 37 libraries (from 32 different samples) obtained from the whole biopsy (Not Sorted sample, NS) and from 4 distinct flow-sorted populations (pre-germinal center (PGC) (IgD+CD38+) centroblasts (CB) (IgD+CD38+CD10+CXCR4+), centrocytes, (CC) (IgD+CD38+CD10+CXCR4-) and memory enriched (ME) (IgD-CD38-) were prepared (S2 Table and Fig 2). As the conventional CB marker CD77 has been reported as an unreliable discriminator of CB and CC [22, 23] we used instead the more recently described marker CXCR4 [23, 24]. To confirm the ability of CXCR4 to distinguish between CB and CC we performed a cell cycle analysis on cells suspensions from tonsils and we observed a significant higher number of cells cycling (S/M phase) in the CB sub-population (IgD-CD38+CD10+CXCR4+) as compared to the CC (IgD-CD38+CD10+CXCR4-) (p = 0.01) (S2C Fig); in addition, by real Time Quantitative PCR, we detected an average 7 fold increased level of AID transcript in the CB and 6.5 fold increase of *mir155h6* in CC from tonsils and FL cell suspensions (S1D Fig), confirming an enrichment of the 2 sub-populations. The staining of the ME (IgD-CD38-) sub-population with the conventional human marker for memory cells CD27 showed that more than 50% of these cells expressed this marker (S1B Fig). No association was observed between the percentage of flow-sorted CB and the histological grade of the biopsies (Table 1).

**Fig 3:**
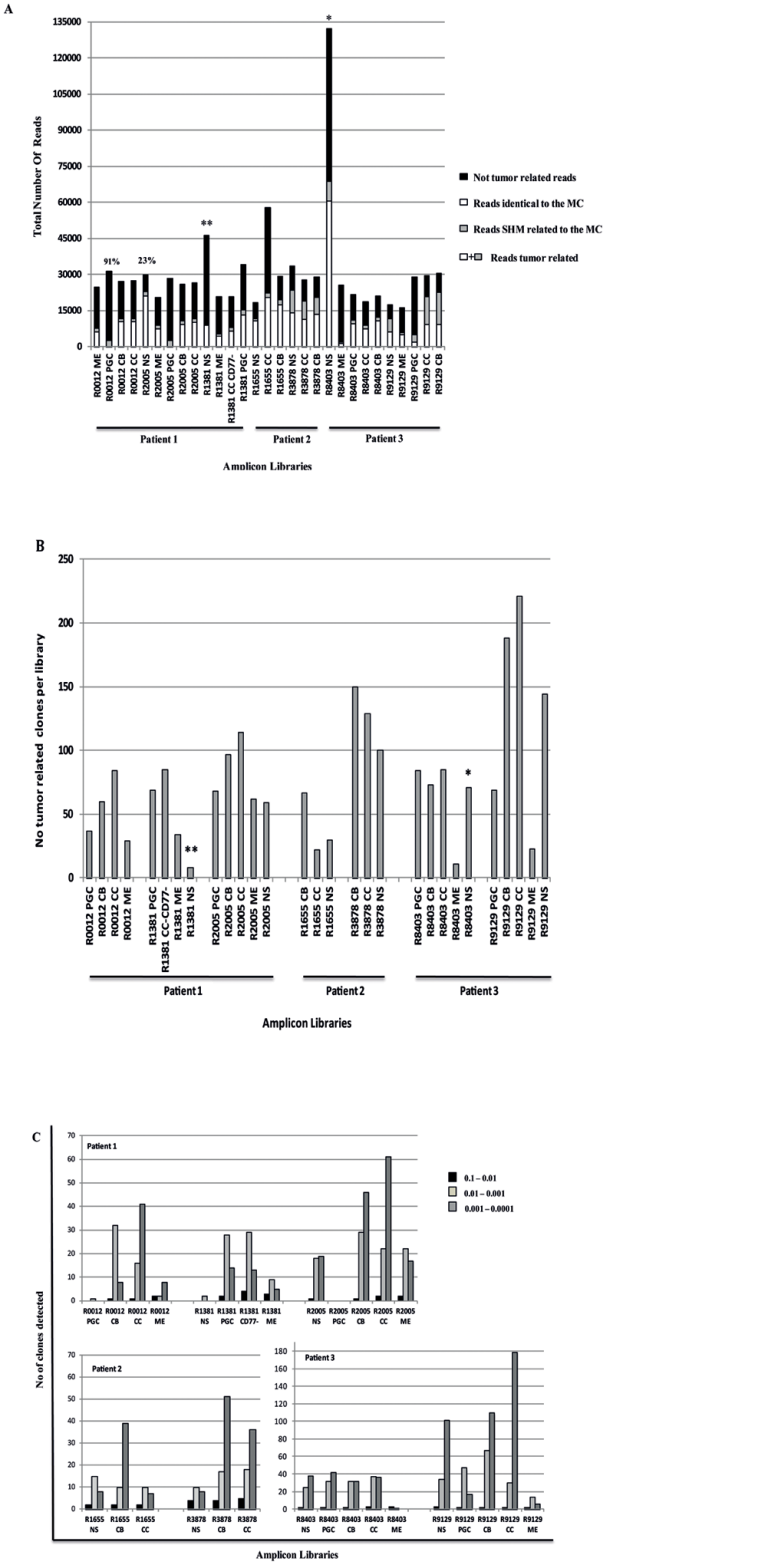
High throughput sequencing results and analysis of the tumor related clone. (A) Histogram showing the tumor related reads generated in the 29 different sub-sets that passed the filtering process. In black are shown the not tumor related reads, generated using VH3 family primers (S1 Table), in white the reads identical to the MC and in grey reads that are clonally related to the MC. * indicates the library prepared performing 6 independent PCRs, using the same primer whilst ** prepared by performing 2 independent PCR and using 2 different MID primers (B) Histogram showing the number of MC related subclones generated per library. * symbol indicates the library prepared by performing 6 independent PCRs with the same primer whilst ** symbol refers to the library prepared by performing 2 independent PCR and using 2 different MID primers. (C) Histogram showing the frequency of the clones per library. The black squares represent the number of clones detected at a frequency between 0.1 and 0.01, the dark grey squares the clones detected at a frequency between 0.01 and 0.001 and the light grey squares the number of clones detected at a frequency between 0.001 and 0.0001.

Our sequencing approach produced ≈ 937,000 sequences (average per library: ≈ 31,200; range 16,372–132,147). Good quality sequences were generated in 35 of these libraries whereas two (R20012NS and R0012CC CD77-) failed the filtering process (S2A Fig). As expected, the highest number of reads was obtained from the R8403NS library prepared performing 6 independent amplifications. To ensure detection of all tumor subclones we utilized VH3 family oligonucleotides rather than tumor specific primers; therefore we observed a heterogeneous level of contaminating sequences from normal B cells ranging between 23% (R2005NS) to 91% (R0012PGC) (Fig 3A). We did not find any correlation between the amount of DNA (or number of cells) used in the preparation of each library and the final number of tumor related reads generated (Spearman correlation coefficient r = -0.3, p = NS) or the numbers of clones detected (Spearman correlation coefficient r = -0.2, p = NS). These data suggest that the diversity observed reflects the load of tumor infiltrating cells and is not a consequence of errors introduced during the preparation of the libraries or the pyrosequencing process.

### Analysis of tumor related clones

Tumor related clones were observed in the CB and CC sub-populations but also in the PGC and ME sorted cells (S3 Table); this result is consistent with the presence of CD10 positive cells (since CD10 is a standard immunodiagnostic marker of FL) not only in the GC compartment but also in the PGC and ME sorted populations (S1E Fig). Clones identical to the MC represented ≥ 70% of the tumor related sequences in 69% (20/29) of the amplicon libraries, 40–70% in 24% (7/29) and less than 40% in the remaining 7% (2/29) (S2B Fig). Compared to the MC sequences, we detected 812 clones in pt1 (13 libraries), 500 in pt2 (6 libraries) and 975 in pt3 (10 libraries) (Fig 3B). Subclones could be detected down to a frequency of 5x10^-3^ (calculated as number of reads identified in a library versus total number of tumor related reads). The prevalence of individual subclones varied by several logs across the libraries (range 10^−1^/10^−4^) and, with the exception of R8403ME, more than 80% were present at a frequency less than 10^−2^ (Fig 3C). We observed a certain degree of inter-patient variability regarding the type of clones detected (e.g. more germline related sequences in the PGC populations of pt1 and in the ME library of FL1 R8403 from pt3) and the region targeted by the SHM machinery (35% of subclones identified in pt1 displayed SHM in the DH-JH region versus only 6% in pt3), possibly reflecting the different amount of tumor infiltrating cells and the time the biopsies were collected (S4 Table).

There were a high number of distinct subclones (range 27% for pt1 to 55% for pt3) shared among libraries from the same biopsy. By looking specifically at these shared and unique reads we observed that in pt1 the majority of these clones resided in the more mature B cell populations CB/CC/ME whilst in pt3 these belonged to the PGC/CB/CC sub-populations (S4A–S4G Fig). These features suggest that the subclones detected in pt1 may be enriched in a more differentiated tumor population, trapped in the GC for longer or alternatively that had been re-circulating several times through the same lymph node.

The dominant MC was detected in sequential biopsies from all cases. The sequential biopsies obtained from pt1 displayed the same MC in all samples investigated and showed 17 subclones, more or less mutated compared to the MC, that co-segregate with the dominant clone (total prevalence ranging from 0.0001 to 0.16) However we could not detect a consistent pattern of clonal evolution, with 7 clones more prevalent in the first biopsy (R0012-tFL), 6 more prevalent in the second (R1381 FL1) and 4 more prevalent in the third biopsy (R2005 FL2)(S5 Table). On the contrary for pt2 we detected the MC from FL1 R1655 in the NS and CB libraries from FL2 R3878 (frequency 0.0004 and 0.0026 respectively) and the MC FL R3878 in the FL1 R1655 NS and CB (frequency 0.0002 and 0.0015). When we compared CB and CC libraries from FL1 and FL2 samples from pt2 we detected 5 additional clones apart the 2 MC that were shared between the 2 biopsies (prevalence range 0.0003–0.0688) The prevalence for all these sub-clones was higher in the second biopsy (S5 Table). In pt3 we identified in 3/5 libraries from the FL1 R8403 samples (PGC, ME and NS) an identical clone to FL2 R9129 MC and one with 1 additional SHM at a frequency between 0.0003 and 0.002. In addition we observed in 4/5 libraries from FL2 R9129 (PGC, CC, ME and NS) a clone identical to the FL1 R8403 and one with 1 more SHM (in NS) at a frequency ranging between 0.003 and 0.007. These results fit with the hypothesis that several minor subclones have the capacity to evade treatment and repopulate the tumor bulk at relapse or transformation.

### Lineage trees analysis

**Fig 4:**
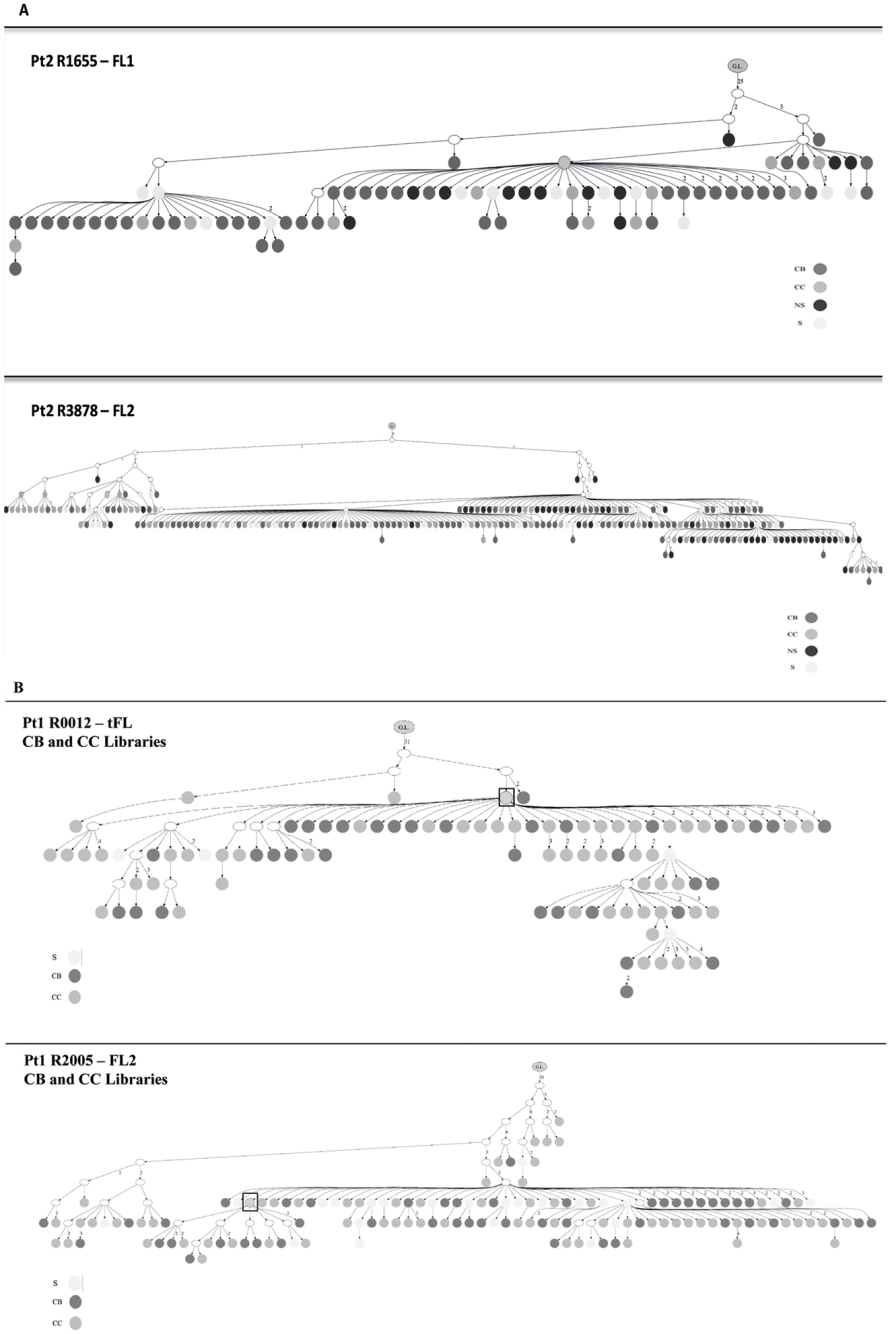
Merged Lineage Trees showing the dynamics of evolution of the sub-clones. (A) Merged lineage trees generated from the 2 biopsies R1655 FL1 and R3878 FL2 from pt2. G.L. means germline; the dark grey circles represent the CB clones, the medium grey circles the CC, the light grey circles the clones shared (S) among 2 or more populations and the black circles those from the not sorted (NS) whole biopsy. (B) Merged lineage trees obtained from the combination of the CB and CC libraries from the samples R0012 t-FL and R2005 FL2 from pt1. The dark grey circles represent the CB subclones, the medium grey circles the CC and the light grey circles the clones shared (S) by CB and CC. The white circles represent subclones not detected with the 454 sequencing. Squares highlight the MC.

To understand the dynamics associated with the maturation of lymphoma cells in secondary lymphoid organs we reconstructed lineage trees from sequence data obtained from individual and from combined libraries; these latter trees, denoted merged trees, were generated using reads detected in more than one sub-population and in the whole tumor sample (Fig 4A and 4B and S5A–S5D Fig).

We observed lineage trees with long trunks (for merged trees, average 23, range 19–31, from samples FL1 R8403 and tFL R0012 respectively), suggestive of the existence of a B cell that had already undergone several rounds of mutation and differentiation before becoming FL and compact but wide branches indicative of an oligoclonal FL population trapped in a narrow stage of maturation, capable of undergoing SHM but unable to further differentiate. Because of the heterogeneity observed in the PGC and ME sub-populations from pt1 and pt3 (S4 Table) and due to the lack of PGC and ME libraries from pt2, we decided to focus our analysis on the CB, CC and NS compartments. There seemed to be no difference in the architecture of the trees and the distribution of the subclones among CB, CC and NS individual trees from the same patient. In all samples investigated we observed a rise in the level of complexity when merged trees were generated. Indeed, whilst in the lineage trees generated from individual libraries the MC maps upstream to the majority of the minor subclones, in the merged trees this clone may move to the bottom of the tree and have a reduced number of descendants (Fig 4B, S5A–S5C Fig). Therefore, it appears that an understanding of the clonal architecture is improved by an increased number of sequences.

When merged trees from individual biopsies were drawn the CB and CC sub-clones did not segregate in different branches or sides of the lineage trees but were randomly distributed (Fig 4B and S5C Fig). We did not observe any difference between merged trees generated from the GC (CB/CC) populations from t-FL and FL samples from pt1 (Fig 4B), or when we compared the merged trees from pt1 (showing a direct evolution pattern and only post-treatment samples included in the analysis) with those from pt3 (displaying a CPC pattern and 2 pre-treatment samples) (S5B Fig). These results suggest that these features are intrinsic to the FL B cell population.

Pt2 displayed a pattern compatible with a direct evolution when analyzed by the heteroduplex analysis, whereas analysis of the merged tree using subclones generated by high throughput sequencing from both biopsies clearly showed a pattern more consistent with a CPC, with the two MC clones mapping on two separated branches and few clones from the FL2 R3878 sample mapping on the same branch of FL1 R1655 clones (S4D Fig). Pt3 also showed a CPC profile, but in this case the two dominant clones (with their corresponding minor subclones) mapped on 2 distant forks of the trees (upstream in the FL1 R8403 and downstream in the FL2 R9129). Interestingly, it was not possible to identify clones with a sequence identical to or with a pattern of SHM similar to the inferred CPC in any of the libraries from these 2 patients.

### Genome wide SNP array of sequential biopsies

**Fig 5:**
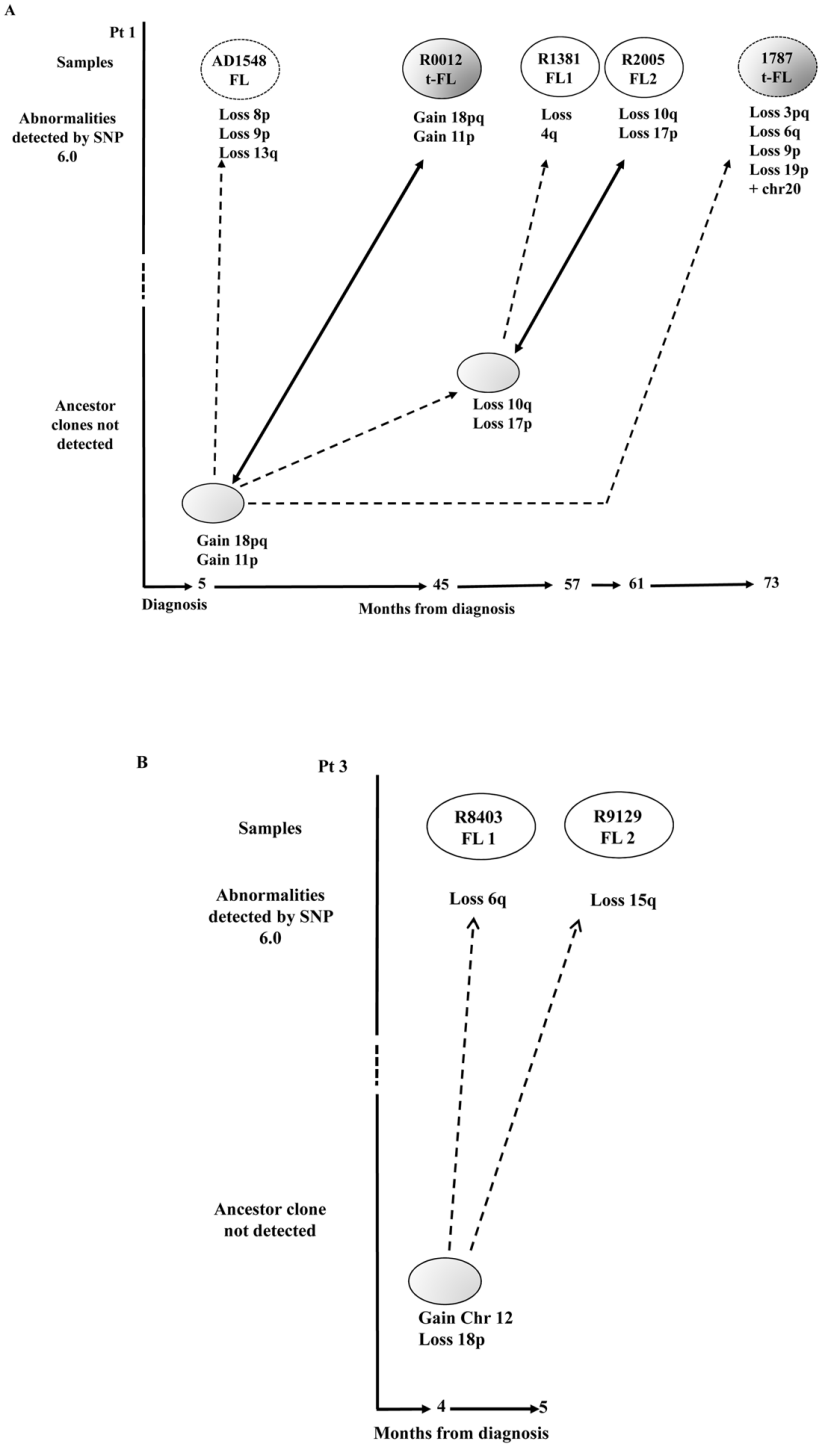
Models of clonal evolution developed by the CNV analysis. FL and t-FL samples are represented in white and dark grey ovals respectively. The CNVs occurring at each stage are shown below the ovals. (A) In pt1 two additional samples (AD1548 FL and 1787 t-FL) that were not investigated during the 454 deep sequencing experiments were analyzed and are shown as dotted ovals. We observed a model of clonal selection, where more primitive ancestor cells not detected in the first biopsy (depicted in light grey) later become the R0012 t-FL and R2005 FL dominant clones. The time course between clinical episodes (bottom) indicates that these precursor cells can persist for many months before disease presents clinically. The dotted arrows represent the evolution from an ancestor subclone into a more mutated tumor cell, whilst the bold arrows show the emergence of an ancestor cell, by clonal selection, identical to the R0012 t-FL and R2005 FL respectively. (B) In pt3 we observe a model of divergent evolution. The more primitive progenitor cell (light grey oval) has already acquired 2 CNV and subsequently acquires the loss of 6q and the loss of 15q arms captured within the R8403 FL1 and R9129 FL2 tumors respectively.

The analysis of genomic copy number variation (CNV) represents an alternative method that our group [16] and others [17] have used to trace the evolution of tumor clones associated with FL at relapse or transformation. To test whether the CNV pattern was concordant with the profile detected by IgH-VH deep sequencing and in order to identify genomic events characteristic of the FL/t-FL samples, a genome wide analysis using the Affymetrix SNP 6.0 array was performed on the FL/t-FL samples from pt1 and pt3. For pt1 two additional samples (AD1548 and 1787) were also included (Figs 1A and 5A). In pt1, who displayed a pattern compatible with a direct evolution, we observed two samples (t-FL R0012 and FL R2005) with fewer CN changes as compared to the immediate preceding biopsy and identical to those of predicted progenitor clones (Fig 5A); this non-linear CPC pattern is consistent with a model of clonal selection and assumes the presence of subclones that persist over time and may become dominant under certain selection pressures such as therapy. Instead, the SNP 6.0 array results of pt3 confirm the existence of a CPC with 2 CNV gains of the entire chromosome 12 and loss of 18p (Fig 5B). This CPC subsequently acquired a further loss of 6q detected in the R8403 FL biopsy and a loss of 15q in the R9129 FL biopsy.

## Discussion

FL is an indolent disease characterized by the clonal expansion of mature B cells from GC of secondary lymphoid organs. Several studies of sequential biopsies from the same patient [6, 7, 14, 15, 17, 25, 26] and two published BMT models [14, 18] revealed the existence of at least two patterns of clonal evolution: direct, through the acquisition of additional mutations over time, and divergent, in which a less differentiated CPC gives rise to different tumor clones [14, 27]. Recent published NGS studies have established that mutations in several genes involved in histones modification [8] play key roles in the early stages of clonal evolution of FL, but little is known regarding the degree of entanglement of FL cells and the dynamics of emergence and disappearance of tumor related variants. Here, for the first time, we attempt to micro-dissect the complexity of FL clones, investigating whole biopsies and selected B cells belonging to different stages of maturation from sequential FL/t-FL samples, using IgH-VH as marker and the 454 sequencing technology.

As in other cancers [20, 28], this technology has proven to be very effective in detecting rare variants. Indeed, by simultaneously investigating the whole biopsy and selected sub-populations we increase significantly the number of unique tumor related clones detected per biopsy, typically at a frequency below 10^−2^ (Fig 3C). The presence of such a large number of variants, all stacked in a dynamic environment such as the GC, has important biological/clinical implications since many of these minor subclones, if maintained, are preordained to acquire additional mutations increasing the risk of subsequent relapse or transformation. Despite the high number of subclones detected in the different sub-populations, we were not able to linearly stratify the tumor clones according to the conventional B cell differentiation pattern. In particular, when we focused on the SHM profile of the PGC and the ME compartments from pt1 and pt3 we observed a high level of heterogeneity across the samples, in terms of homology to the germline VH gene and number of SHM in the DH-JH region, across the different samples (S4 Table). A possible explanation for such variability may be the lack of expression of the conventional B cell markers on the surface of tumor cells, the use of CD27 and IgD to sort PGC and ME sub-populations, or a possible cross-contamination during the flow-sorting.

The investigation of the IgH-VH SHM patterns and clonal relationships provided insight into the transition from normal to malignant B cells. We observed lineage trees with a structure consistent with a long mutational history and high intraclonal diversification. A qualitative analysis of merged trees generated using CB and CC subclones from the same biopsy showed that these two populations do not map on separated branches or sides of the same lineage tree but instead are mixed and scattered throughout. The identification of increased expression of *AICDA* in the CB and *mir155h6* in the CC compartments, in combination with the detection of a greater percentage of cycling cells in the CB compartment (S1C and S1D Fig) suggest that any potential contamination between the 2 compartments is at a level below the sensitivity of the RQ-PCR assay and confirms previous published data, generated using a multiphoton laser-scanning microscopy technology that show a continuous interplay between these two sub-populations. [29, 30] The detection of an increased number of CD10 positive cells in both the PGC and ME compartments from FL samples, as compared to tonsils and t-FL (S1D Fig) is consistent with the detection of tumor clones within all our sorted populations and fits with the recent mouse models describing B memory cells being able to circulate and disseminate not only in peripheral blood but also in lymphoid organs.[31]

The simultaneous comparison of sequential biopsies from the same individual and from different patients is crucial for understanding the patterns of clonal evolution. Of note, we observed no difference in the shape of the lineage trees or distribution of the subclones across the libraries, suggesting that precursor lymphoma B cells continue to follow the dynamics of non malignant B cells, in all the FL and t-FL samples analyzed.

Despite the elevated number of unique variants detected across all the libraries we were not able to identify a sequence compatible with the putative CPC. Our group and others [14, 17] have previously suggested that such cells may be present either below the levels of detection or may have left the lymph node entirely. The first model is compatible with the pattern observed in pt2. In this case the merged lineage trees showed that the two tumors were related by an ancestor cell that was not itself detected during the 454 sequencing. Since both biopsies were excised from the same location (left inguinal biopsy) and the two MCs displayed a closely related pattern of SHM, we may hypothesize that this CPC still co-exists within the tumor populations, but at a level below even the sensitivity of detection here. The presence of 5 sub-clones apart the two MC in both the biopsies, at higher prevalence in the second biopsy (S5 Table) suggests that other clones, apart the MC are capable of surviving treatment. The second model perfectly depicts the scenario observed in pt3. In this case the biopsies were excised from two distinct lymph nodes (right axilla, FL1-R8403 and right femoral node, FL2-R9129) (Table 1). Merged lineage trees clearly showed that the MC and tumor related clones from FL1 preceded the FL2 tumor populations and were related by a CPC, that was not detected). However, when we extended the sequence analysis to the DH-JH region and to the tumor specific t(14;18) rearrangement we identified 21 insertions in the FL1 N1 and N2 regions of CDR3 as compared to FL2 R9129, and an identical BCL2/IgH rearrangement, carrying a mutation in the 3’ of BCL2 in the FL1 R8403 (S6A Fig). Together these results are consistent with the existence of a precursor cell that migrated from one lymph node to the other where the two lymphomas developed independently. However by SNP profiling the R8403 and R9129 FL samples we observed two copy number variations in common between the two biopsies (Fig 5B) suggestive of an ancestor that had already acquired some genomic lesions. The inability of this putative cell to proliferate and develop into overt lymphoma is consistent with the latency observed in the BMT model [14, 18] and fits with the model of an immune-system capable of regulating the pace of FL and restraining the expansion of the CPC. [32, 33] The detection of the 2 MCs in both the biopsies (S6B Fig), albeit at very low levels, can be explained by the ability of FL cells to leave the original GC and migrate to other lymphoid organs.

Interestingly the pattern of evolution observed in pt1 was more complex and the sequencing strategy revealed a direct evolution pattern, unraveling the existence of an identical common dominant clone in each of the biopsies tested (t-FL R0012, FL1 R1381 and FL2 R2005) amongst a pool of 17 additional clones, either more mature or immature compared to the MC. Conversely, the SNP 6.0 genotyping data supported a CPC pattern compatible with the selection of subclones over a period of 6 years that were not detected in the first biopsy investigated, but, according to the CNV data, were inferred to have existed in earlier biopsies. These apparently discordant results may reflect the different sensitivity of the techniques, with IgH-VH profiling, the complexity of the tumor populations following the physiological mechanism of SHM of the *IgH* gene and the SNP 6.0 array depicting a snapshot of clonal genetic aberrations present in the whole biopsy. It is not yet clear whether clones defined according to the pattern of SHM share any of the lesions observed by SNP profiling and could represent the rare variant not detected at diagnosis becoming the dominant clone at relapse and transformation. Recently by using a genome sequencing approach and simultaneously analyzing different genetic mutations detected in paired FL-tFL samples, our group has shown the existence of a CPC pattern in all the samples investigated and re-defined the patterns of evolution observed as a "sparse" CPC with very little mutations shared by FL and t-FL and a "rich" CPC, with high clonal resemblance between the two paired samples [6].

The high-throughput sequencing analysis of the IgH-VH gene performed on different sorted sub-populations corresponding to different stages of B cell maturation did not prove to be successful in terms of identification of distinct clonal populations but captured a hitherto unreported level of tumor cell diversity. Our observations demonstrate the existence of an entangled architecture in FL, with different tumor clones able to evolve following diverse patterns. The investigation of different sub-populations showed an increase in the level of intermingling of the tumor related clones but not a change in the lineage trees' architecture. This, in combination with the lack of detection of a CPC, suggests that this precursor (defined by IgH-VH) may have acquired additional SHM and therefore display a more mutated IgH-VH from the one predicted. The comparison of sequential samples has unraveled some rare variants that evade treatments and co-segregate with the MC (17 subclones in pt1 and 5 in pt2). The contribution of these cells to the formation and evolution of FL is still unknown, but as observed by SNP 6.0 profiling of samples from pt1, the possible existence of rare variants, that becomes dominant at relapse or transformation, strengthen the importance of eradicating all tumor related cells at first treatment. The discordant patterns observed in pt1 also highlight the importance of including multiple sequential samples from the same patient and all the somatic mutations detected in the tumor when tracing the pattern of disease clonal evolution. As for other malignancies [34, 35], such genetic heterogeneity within the tumor population may represent a major challenge for the development of a personalized therapy with the need to consider the suitability of actionable mutations on a patient by patient basis. Indeed our data suggests that the clonal complexity observed at one location may differ markedly from the pattern observed at other sites. When tumor B cells from lymph nodes dissected from distinct anatomical regions were compared, we observed an evolution of the lymphoma clones following similar dynamics to that observed when metastatic solid tumors are simultaneously investigated. [36, 37] Future data coming from NGS of selected tumor sub-populations from different anatomical compartments and single cell analysis will allow us to capture the complexity of the genetics of FL in its entirety and offer the possibility to identify and characterize the cell of origin of FL.

## Supporting Information

S1 Fig Flow Characterization of the 4 sub-populations investigated by high-throughput sequencing. (A) Staining with CD20. Cell suspensions from tonsils were stained with the same antibodies used for flow-sorting (IgD, CD38, CD10 and CXCR4) plus CD20-FITC. Data shown are representative of experiments performed on 6 different tonsil samples. IgD-CD38- population 20.5% (range 10–32%); IgD-CD38+ population 4.2 (range 2.0–9.2%); IgD-CD38+CD10+CXCR4+ (range 0.6–14.6%); IgD-CD38+CD10+CXCR4- population 8.6 (range 1.6–14.2%). (B): Staining of the IgD-CD38- sub-population with the antibody CD27. Tonsil cell suspensions were stained for the indicated surface molecules and analyzed by flow cytometry. The antibody IgD-PE was used instead of IgD-APC. Data are representative of 6 different samples. (range 38.8–80.7%) (C) Cell cycle analysis in the CB (IgD-CD38+CD10+CXCR4+) and CC (IgD-CD38+CD10+CXCR4-) subsets, defined using IgD-FITC, CD38-PECy7, CD10-PE and CXCR4-APC antibodies. The percentage of cells in the S/M phase is representative of 3 independent experiments and p was calculated using t-test. The flow-cytometry analysis was performed with FlowJo software. (D) Expression of AID (on the top left) and microRNA-155 (on the top right) in the CB (CXCR4+) and CC (CXCR4-) GC subpopulations from patients and tonsils. Results are representative of 5 different FL and 3 tonsils cell suspensions samples for AID and of 4 FL and 3 tonsils for microRNA155. Results of Real Time PCR analysis are expressed relative to gene expression detected in cells from the lymphoblastoid cell line NcNc and as a fold changes (at the bottom), calculated as fold change expression of AID transcripts from CB *vs* CC and microRNA155 transcripts detected in CC vs CB. As expected, AID and microRNA155 are inverted regulated in the CB and CC populations, with AID (*AICDA*) being 7 times more expressed in the CB compartment and microRNA155 (*mir155h6*) being 6.5 times more expressed in the CC sub-set. (E) Percentage of CD10 positive cells in tonsils, FL and t-FL samples. An increased percentage of CD10 positive cells was detected in the PGC (Kruskal-Wallis test p = 0.049, Dunn’s Multiple Comparison Test p = NS) and in the MEsub-populations (Kruskal-Wallis test p = 0.0117, Dunn’s Multiple Comparison Test: tonsils vs FL p<0.05, tonsil vs t-FL and FL vs t-FL = NS). The analysis was performed on cell suspensions from tonsils (7 samples), FL (6 samples) and t-FL (4 samples).
(ZIP)

S2 Fig Total reads and tumor related reads. (A) Histogram of the number of reads generated per library; in black are shown all the reads generated with Roche 454 GS-FLX Titanium and in white the corresponding sequences that passed the filtering control. (B) Histogram showing the percentage of tumor related reads (defined as all the reads identical to the sequence of the dominant clone plus those that according to the SHM pattern are clonally related) per library. The two dotted arrows separate the libraries in 3 different groups: first group, with MC making up for >80% of tumor related reads, second between 40–80% and last group with MC reads being <40% of total tumor reads.
(TIF)

S3 Fig Flowchart of the different steps involved in the generation of the final reads included in the analysis. (TIF)

S4 Fig Schematic representation of the shared and unique clones detected in the NS and sorted sub-populations from pt3 and pt1. (A) R8403 NS sample, (B) R8403 sub-populations, (C) R9129 NS, (D) R9129 sub-populations, (E) R0012 sub-populations, (F) R2002 NS sample and (G) R2005 sub-populations. The pie graphs show the percentage of unique clones (dark grey), clones shared with the NS sample (medium grey) and clones shared with other sub-populations from the same biopsy (light grey). The Venn diagrams depict number of unique and shared clones between two, three, four or five (in the case of the NS sample) different libraries. According to the Venn diagram we observed that the majority of clones in the samples from pt3 belonged to the PGC, CB and CC subpopulations, whilst those from pt1 to the CB, CC and ME sub-sets. When we evaluated the prevalence of shared sub-clones within a sub-population we could not discriminate a consistent pattern of evolution.
(TIF)

S5 Fig Merged Lineage trees. (A) Lineage trees generated from the R2005 FL2 biopsy from pt. G.L. means germline; the dark grey circles represent the CB clones, the medium grey circles the CC, the light grey circles the clones shared (S) among 2 or more populations and the black circles those from the not sorted (NS) whole biopsy. The square depicts the MC. (B) Lineage trees generated from the different libraries of R8403 FL1 and R9129 FL2 from pt3. On the top, the dark grey circles represent the CB subclones, the medium grey circles the CC, the light grey circles shared clones and the black circles the clones detected in the PGC and NS libraries. The squares depict the MC from the biopsy while the dotted rectangles the MC and the clones identical to the MC plus 1 SHM from the other biopsy (C) Merged lineage trees obtained from the CB and CC libraries from the sample R8403 FL1 from pt3. The dark grey circles represent the CB subclones, the medium grey circles the CC and the light grey circles the clones shared (S) by CB and CC. (D) Merged lineage trees from not sorted NS libraries from the samples R1655 FL1 and R3878 FL2 from pt2. The dark grey circles represent the shared clones (S), the medium grey circles the R3878 NS clones, the light grey circles the R1655 NS clones and the black circles the 2 MCs. The white circles represent subclones not detected with the 454 sequencing. Squares highlight the MCs samples.
(TIF)

S6 Fig Characterization of the clones detected in pt3. (A) Alignment of the CDR3 region of the IgH-VH3 MC reads from samples R8403 and R9129 from pt3. (B) Comparison of the MBR *BCL2/IgH* rearrangement from samples R8403 and R9129. (C) Frequency of detection of the R8403MC in the libraries from R9129 and vice versa of the R9129MC in the libraries from R8403.
(TIF)

S7 Fig Alignment of the MC sequences from pt1 and pt2. Sequences, detected by HH analysis, were aligned using ClustelW2. The alignment of the MCs sequences from the samples R0012 t-FL, R1381 FL1 and R2005 FL2 from pt1 (at the top) showed an identical dominant clone in all the 3 biopsies. The alignment of the MC sequences from R1655 FL1 and R3878 FL2 samples from pt2 instead (at the bottom) showed 3 bases mutated. Because all these 3 mutations were also different from the germline sequence (germline bases showed in bold on the top) and fall in a hotspot region (data not shown) it is possible that the same clone is mutated twice in the same bases. In both these cases therefore the presence of a pattern of direct evolution cannot be ruled out.
(TIF)

S1 Table Additional clinical information regarding the 3 patients investigated. (DOC)

S2 Table List of samples and sequence of the primers used for the high-throughput sequencing. (DOC)

S3 Table Number and % of clones detected in the NS and sorted populations. (DOC)

S4 Table Characteristics of the PGC and ME sub-populations. (DOC)

S5 Table Prevalence of the haplotypes shared among the Pt1 samples and Pt2 samples. (DOC)
