# Supplementary figures and images for: High Throughput Sequencing Analysis of the Immunoglobulin Heavy Chain Gene from Flow-Sorted B Cell Sub-Populations Define the Dynamics of Follicular Lymphoma Clonal Evolution
Source: PLoS One. 2015 Sep 1;10(9):e0134833. doi: 10.1371/journal.pone.0134833 (PMC4556522; doi:10.1371/journal.pone.0134833)

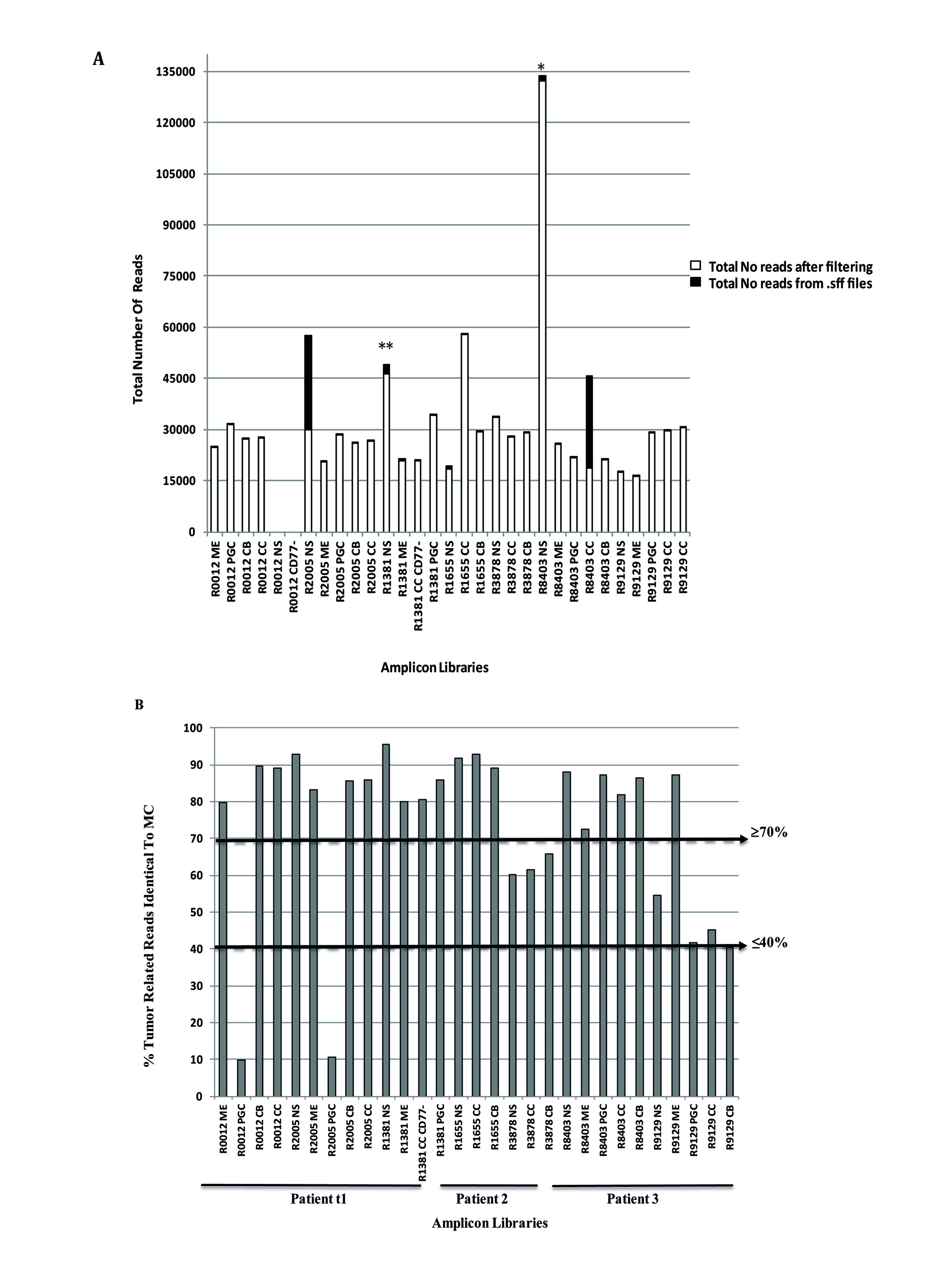

Supplement: S2 Fig — (A) Histogram of the number of reads generated per library; in black are shown all the reads generated with Roche 454 GS-FLX Titanium and in white the corresponding sequences that passed the filtering control. (B) Histogram showing the percentage of tumor related reads (defined as all the reads identical to the sequence of the dominant clone plus those that according to the SHM pattern are clonally related) per library. The two dotted arrows separate the libraries in 3 different groups: first group, with MC making up for >80% of tumor related reads, second between 40–80% and last group with MC reads being <40% of total tumor reads. (TIF) [file pone.0134833.s002.tif]

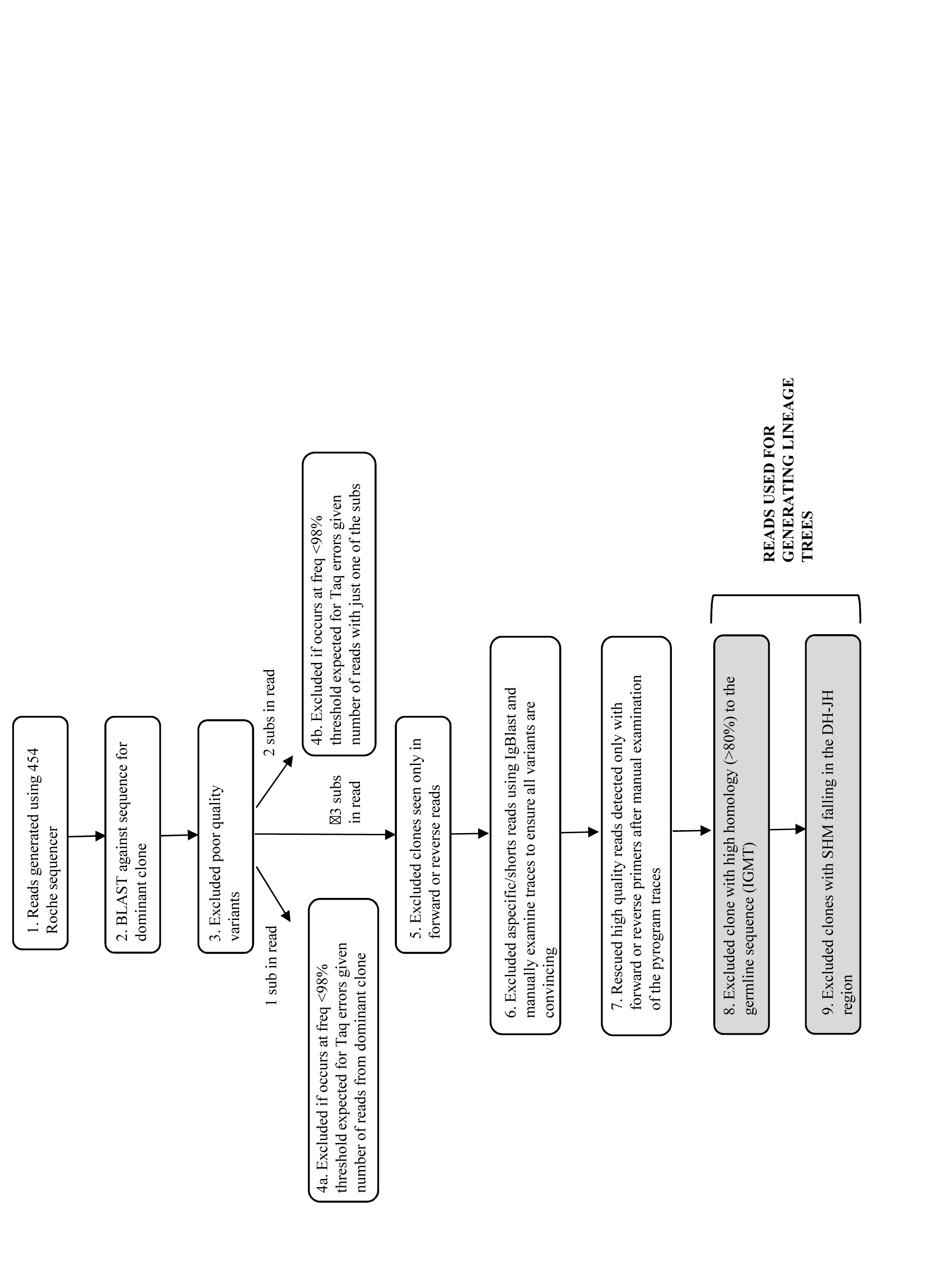

Supplement: S3 Fig — (TIF) [file pone.0134833.s003.tif]

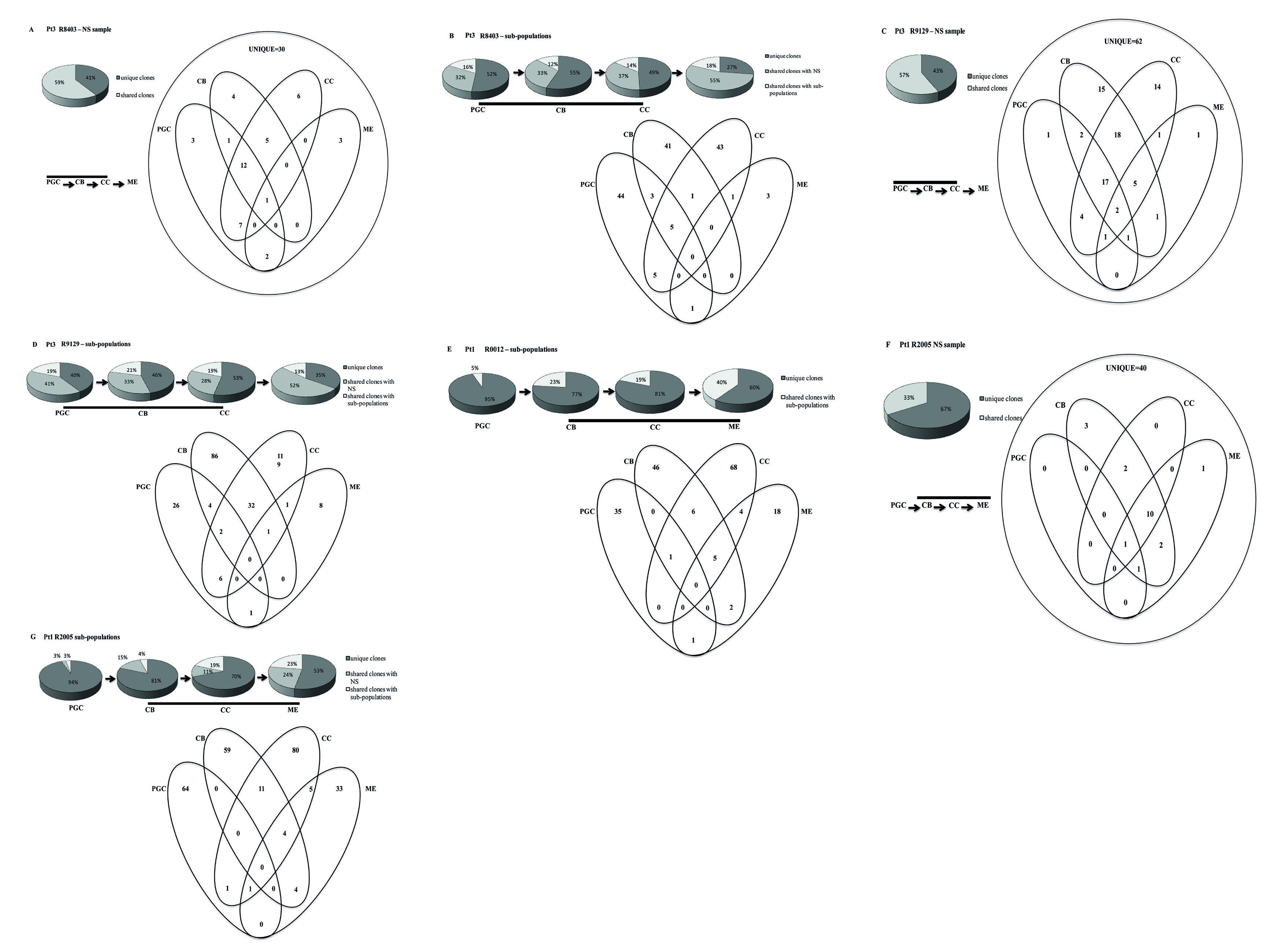

Supplement: S4 Fig — (A) R8403 NS sample, (B) R8403 sub-populations, (C) R9129 NS, (D) R9129 sub-populations, (E) R0012 sub-populations, (F) R2002 NS sample and (G) R2005 sub-populations. The pie graphs show the percentage of unique clones (dark grey), clones shared with the NS sample (medium grey) and clones shared with other sub-populations from the same biopsy (light grey). The Venn diagrams depict number of unique and shared clones between two, three, four or five (in the case of the NS sample) different libraries. According to the Venn diagram we observed that the majority of clones in the samples from pt3 belonged to the PGC, CB and CC subpopulations, whilst those from pt1 to the CB, CC and ME sub-sets. When we evaluated the prevalence of shared sub-clones within a sub-population we could not discriminate a consistent pattern of evolution. (TIF) [file pone.0134833.s004.tif]

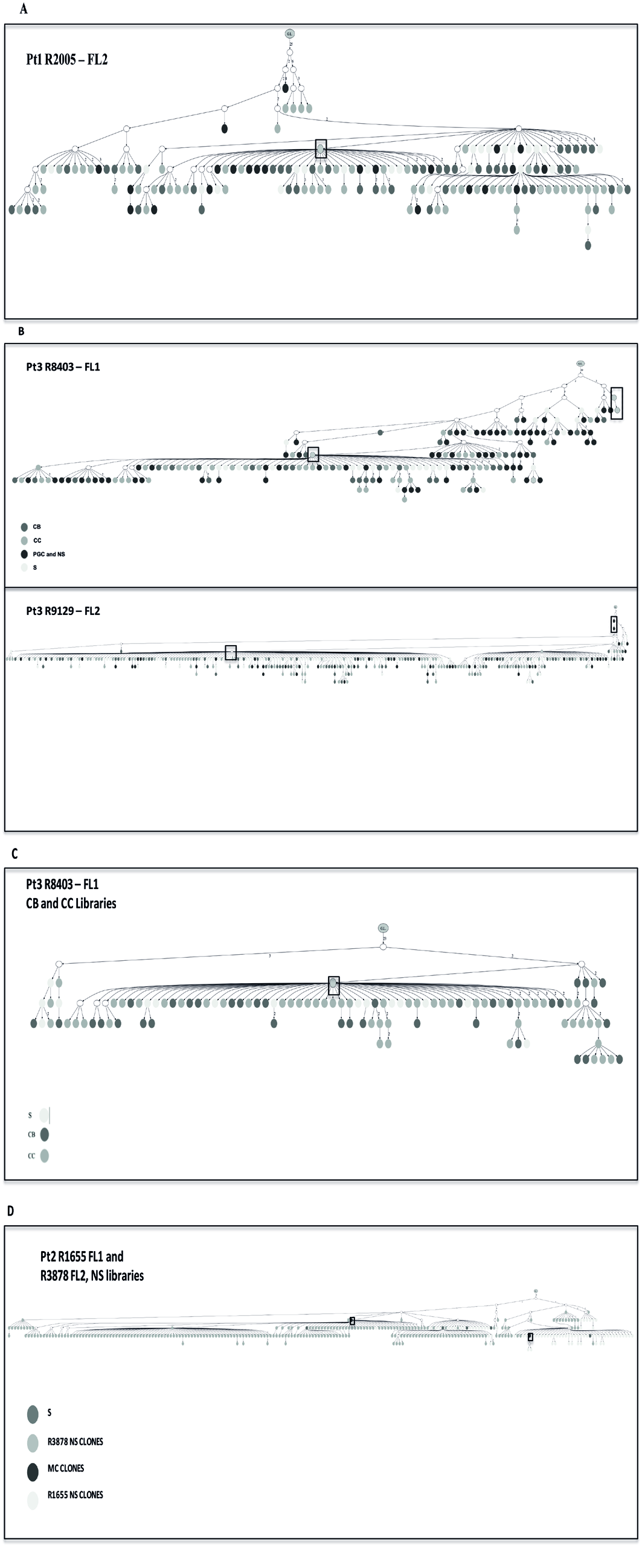

Supplement: S5 Fig — (A) Lineage trees generated from the R2005 FL2 biopsy from pt. G.L. means germline; the dark grey circles represent the CB clones, the medium grey circles the CC, the light grey circles the clones shared (S) among 2 or more populations and the black circles those from the not sorted (NS) whole biopsy. The square depicts the MC. (B) Lineage trees generated from the different libraries of R8403 FL1 and R9129 FL2 from pt3. On the top, the dark grey circles represent the CB subclones, the medium grey circles the CC, the light grey circles shared clones and the black circles the clones detected in the PGC and NS libraries. The squares depict the MC from the biopsy while the dotted rectangles the MC and the clones identical to the MC plus 1 SHM from the other biopsy (C) Merged lineage trees obtained from the CB and CC libraries from the sample R8403 FL1 from pt3. The dark grey circles represent the CB subclones, the medium grey circles the CC and the light grey circles the clones shared (S) by CB and CC. (D) Merged lineage trees from not sorted NS libraries from the samples R1655 FL1 and R3878 FL2 from pt2. The dark grey circles represent the shared clones (S), the medium grey circles the R3878 NS clones, the light grey circles the R1655 NS clones and the black circles the 2 MCs. The white circles represent subclones not detected with the 454 sequencing. Squares highlight the MCs samples. (TIF) [file pone.0134833.s005.tif]

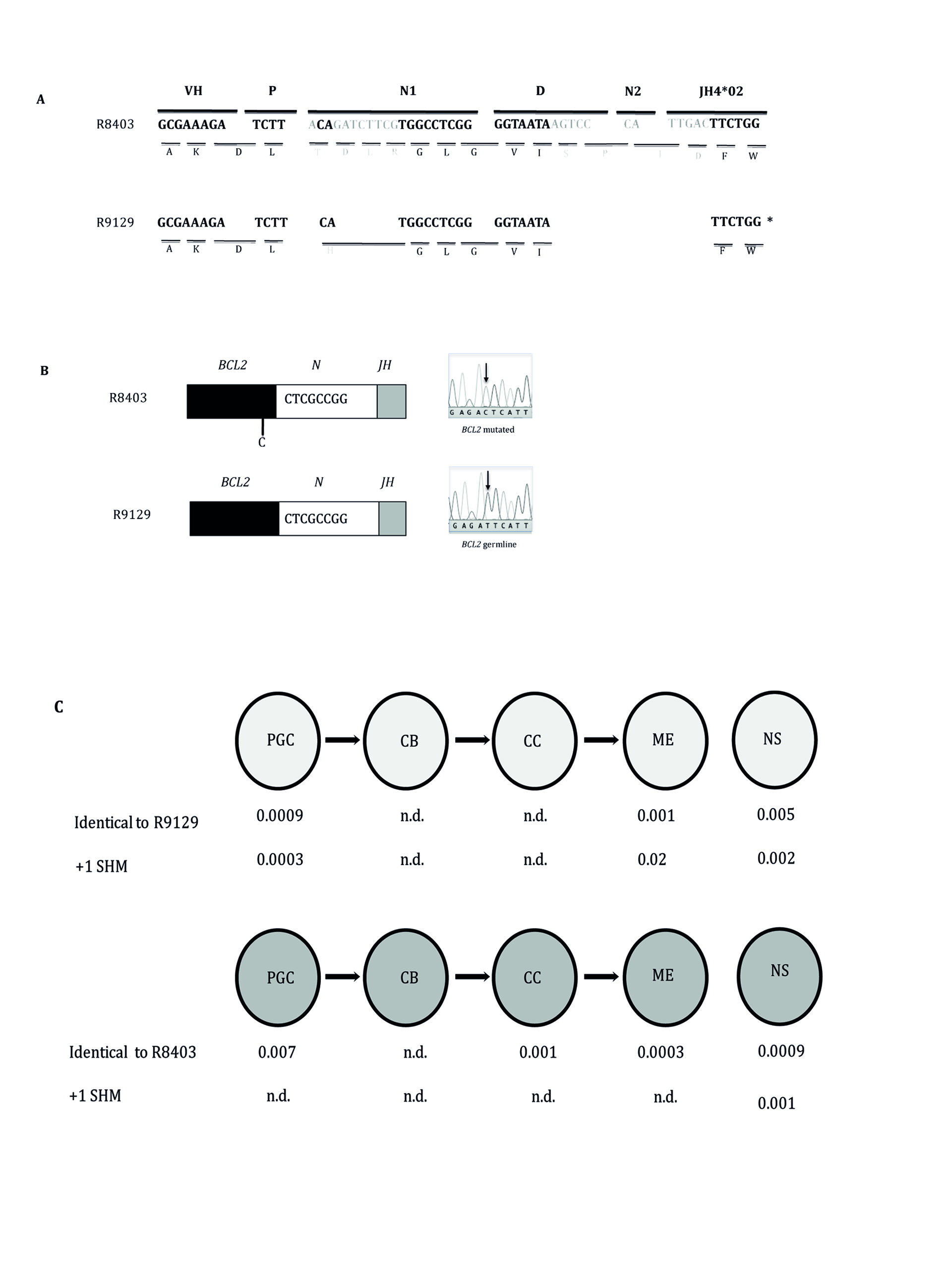

Supplement: S6 Fig — (A) Alignment of the CDR3 region of the IgH-VH3 MC reads from samples R8403 and R9129 from pt3. (B) Comparison of the MBR BCL2/IgH rearrangement from samples R8403 and R9129. (C) Frequency of detection of the R8403MC in the libraries from R9129 and vice versa of the R9129MC in the libraries from R8403. (TIF) [file pone.0134833.s006.tif]

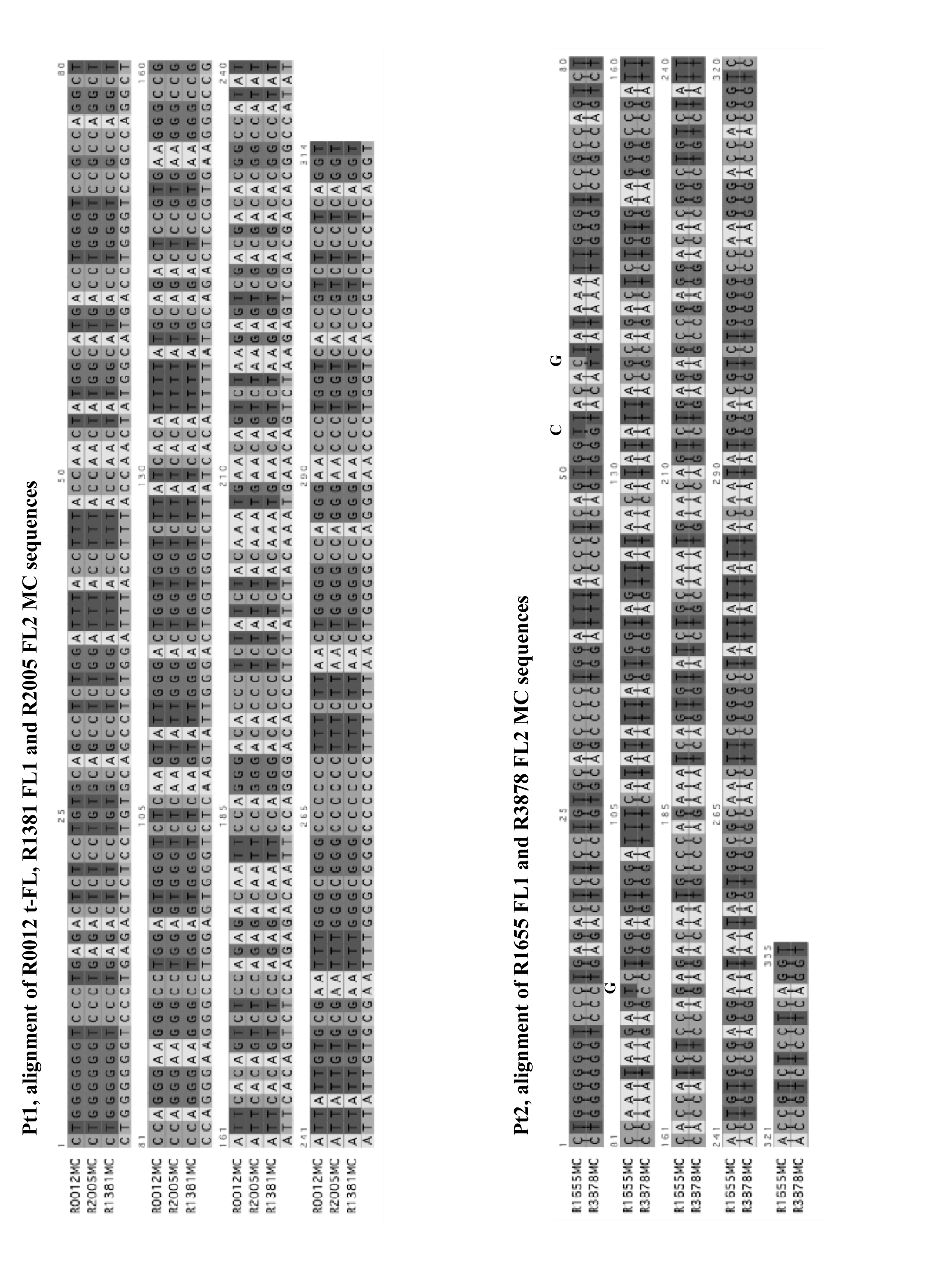

Supplement: S7 Fig — Sequences, detected by HH analysis, were aligned using ClustelW2. The alignment of the MCs sequences from the samples R0012 t-FL, R1381 FL1 and R2005 FL2 from pt1 (at the top) showed an identical dominant clone in all the 3 biopsies. The alignment of the MC sequences from R1655 FL1 and R3878 FL2 samples from pt2 instead (at the bottom) showed 3 bases mutated. Because all these 3 mutations were also different from the germline sequence (germline bases showed in bold on the top) and fall in a hotspot region (data not shown) it is possible that the same clone is mutated twice in the same bases. In both these cases therefore the presence of a pattern of direct evolution cannot be ruled out. (TIF) [file pone.0134833.s007.tif]
