# Supplementary material for: High Throughput Sequencing Analysis of the Immunoglobulin Heavy Chain Gene from Flow-Sorted B Cell Sub-Populations Define the Dynamics of Follicular Lymphoma Clonal Evolution
Source: PLoS One. 2015 Sep 1;10(9):e0134833. doi: 10.1371/journal.pone.0134833 (PMC4556522; doi:10.1371/journal.pone.0134833)
Supplement: S1 Table — (DOC) [file pone.0134833.s008.doc]

**S1 Table Additional clinical information regarding the 3 patients investigated.**

| **Pt No** | **Age at diagnosis**  **(years)** | **No Lines Therapy before 1st biopsy** | **No Lines Therapy before 2nd biospy** | **No Lines Therapy before 3rd biospy** | **No Lines Therapy before 4th biospy** | **No Lines Therapy before 5th biospy** | **FLIPI±** | **Overall Survival**  **Years (months)** | **Time to transformation**  **(years)** | **Overall Survival**  **(years)** |
| --- | --- | --- | --- | --- | --- | --- | --- | --- | --- | --- |
| 1 | 47 | 3# | 5# | 2# | 0# | 1# | N.A. | **11** | 8 | **11** |
| 2 | 43 (7 months) | 5* | 2* | N.A. | N.A. | N.A. | N.A. | 14 (7 months) | 5 | 14 (7 months) |
| 3 | 68 (9 months) | 0 | 0 | N.A. | N.A. | N.A. | Low risk | 9 (2 months) | 4 (8 months) | 9 (2 months) |

N.A. not available

± Pt1 and Pt2 have been diagnosed in a different hospital and therefore FLIPI is not available.

# Pt1 therapy before 1st biopsy consisted of: Chlorambucil + Prednisolone, Chlorambucil, IDEC-C2B8 (Rituximab); before 2nd biopsy of: fludarabine, mitoxantrone, dexamethasone (FMD), Anti-CD20 antibody (B1) combined with I^131^, cyclophosphamide, hydroxy doxorubicin, vincristine, prednisone (CHOP), cyclophosphamide and radiotherapy; before 3rd biopsy of: VP16/Ara-C combination, carmustine, etoposide, Ara-C, melphalan (BEAM) followed by ASCT; before 5th biopsy of: PS341 (Velcade).

*Pt2 Pt1 therapy before 1st biopsy consisted of: CHOP, Fludarabine, Rituximab, Chlorambucil, Bortezomib; before 2nd biopsy of: Interferon, MethylPrednisolone.
