# Supplementary material for: High Throughput Sequencing Analysis of the Immunoglobulin Heavy Chain Gene from Flow-Sorted B Cell Sub-Populations Define the Dynamics of Follicular Lymphoma Clonal Evolution
Source: PLoS One. 2015 Sep 1;10(9):e0134833. doi: 10.1371/journal.pone.0134833 (PMC4556522; doi:10.1371/journal.pone.0134833)
Supplement: S2 Table — (DOC) [file pone.0134833.s009.doc]

**S2 Table List of samples and sequence of the primers used for the highthroughput sequencing**

| **Pt ID** | **Sample ID** | **Tumor** | **Population** | **No libraries**  **Run on 454** | **Forward** | **Reverse** |
| --- | --- | --- | --- | --- | --- | --- |
| 1 | R0012 | t-FL | **Not sorted** | 1 | **AGAT**CTGGGGGGTCCCTGAGACTCTCCTG | **AGAT**ACCTGAGGAGACGGTGACC |
|  |  |  | ME | 1 | **TTAT**CTGGGGGGTCCCTGAGACTCTCCTG | **TTAT**ACCTGAGGAGACGGTGACC |
|  |  |  | **CC CD77-^** | 1 | **CGTTA**CTGGGGGGTCCCTGAGACTCTCCTG | **CGTTA**ACCTGAGGAGACGGTGACC |
|  |  |  | PGC | 1 | **TCTTA**CTGGGGGGTCCCTGAGACTCTCCTG | **TCTTA**ACCTGAGGAGACGGTGACC |
|  |  |  | CB | 1 | **TTTAT**CTGGGGGGTCCCTGAGACTCTCCTG | **TTTAT**ACCTGAGGAGACGGTGACC |
|  |  |  | CC | 1 | **ACGTTA**CTGGGGGGTCCCTGAGACTCTCCTG | **ACGTTA**ACCTGAGGAGACGGTGACC |
|  |  |  |  |  |  |  |
|  | R1381 | FL | Not-sorted± | 1 | **CGTA**CTGGGGGGTCCCTGAGACTCTCCTG | **CGTA**ACCTGAGGAGACGGTGACC |
|  |  |  | Not-sorted± | 1 | **CCTA**CTGGGGGGTCCCTGAGACTCTCCTG | **CCTA**ACCTGAGGAGACGGTGACC |
|  |  |  | ME | 1 | **TATAT**CTGGGGGGTCCCTGAGACTCTCCTG | **TATAT**ACCTGAGGAGACGGTGACC |
|  |  |  | CC CD77-^ | 1 | **TATTA**CTGGGGGGTCCCTGAGACTCTCCTG | **TATTA**ACCTGAGGAGACGGTGACC |
|  |  |  | PGC | 1 | **TTTTA**CTGGGGGGTCCCTGAGACTCTCCTG | **TTTTA**ACCTGAGGAGACGGTGACC |
|  |  |  |  |  |  |  |
|  | R2005 | FL | Not sorted | 1 | **TTTA**CTGGGGGGTCCCTGAGACTCTCCTG | **TTTA**ACCTGAGGAGACGGTGACC |
|  |  |  | CC | 1 | **TCTAT**CTGGGGGGTCCCTGAGACTCTCCTG | **TCTAT**ACCTGAGGAGACGGTGACC |
|  |  |  | ME | 1 | **TGTTA**CTGGGGGGTCCCTGAGACTCTCCTG | **TGTTA**ACCTGAGGAGACGGTGACC |
|  |  |  | PGC | 1 | **ACGTAT**CTGGGGGGTCCCTGAGACTCTCCTG | **ACGTAT**ACCTGAGGAGACGGTGACC |
|  |  |  | CB | 1 | A**TCTTA**CTGGGGGGTCCCTGAGACTCTCCTG | A**TCTTA**ACCTGAGGAGACGGTGACC |
|  |  |  |  |  |  |  |
| 2 | R1655 | FL | Not sorted | 1 | **GCAT**CTGGGGGGTCCCTGAGACTCTCCTG | **GCAT**ACCTGAGGAGACGGTGACC |
|  |  |  | CB | 1 | **CGAT**CTGGGGGGTCCCTGAGACTCTCCTG | **CGAT**CTGGGGGGTCCCTGAGACTCTCCTG |
|  |  |  | CC | 1 | **CGTTA**CTGGGGGGTCCCTGAGACTCTCCTG | **CGTTA**ACCTGAGGAGACGGTGACC |
|  |  |  |  |  |  |  |
|  | R3878 | FL | Not sorted | 1 | **ACAT**CTGGGGGGTCCCTGAGACTCTCCTG | **ACAT**ACCTGAGGAGACGGTGACC |
|  |  |  | CB | 1 | **CGTAT**CTGGGGGGTCCCTGAGACTCTCCTG | **CGTAT**ACCTGAGGAGACGGTGACC |
|  |  |  | CC | 1 | **TGAT**CTGGGGGGTCCCTGAGACTCTCCTG | **TGAT**ACCTGAGGAGACGGTGACC |
|  |  |  |  |  |  |  |
| 3 | R8403 | FL | Not-sorted* | 6 | **CCAT**CTGGGGGGTCCCTGAGACTCTCCTG | **CCAT**ACCTGAGGAGACGGTGACC |
|  |  |  | PGC | 1 | **ACTA**CTGGGGGGTCCCTGAGACTCTCCTG | **ACTA**ACCTGAGGAGACGGTGACC |
|  |  |  | CB | 1 | **TCAT**CTGGGGGGTCCCTGAGACTCTCCTG | **TCAT**ACCTGAGGAGACGGTGACC |
|  |  |  | CC | 1 | **TCTA**CTGGGGGGTCCCTGAGACTCTCCTG | **TCTA**ACCTGAGGAGACGGTGACC |
|  |  |  | ME | 1 | **TGTAT**CTGGGGGGTCCCTGAGACTCTCCTG | **TGTAT**ACCTGAGGAGACGGTGACC |
|  |  |  |  |  |  |  |
|  | R9129 | FL | Not-sorted* | 1 | **GCTA**CTGGGGGGTCCCTGAGACTCTCCTG | **GCTA**ACCTGAGGAGACGGTGACC |
|  |  |  | PGC | 1 | **CTAT**CTGGGGGGTCCCTGAGACTCTCCTG | **CTAT**ACCTGAGGAGACGGTGACC |
|  |  |  | CB | 1 | **ATAT**CTGGGGGGTCCCTGAGACTCTCCTG | **ATAT**ACCTGAGGAGACGGTGACC |
|  |  |  | CC | 1 | **ATTA**CTGGGGGGTCCCTGAGACTCTCCTG | **ATTA**ACCTGAGGAGACGGTGACC |
|  |  |  | ME | 1 | **CTTA**CTGGGGGGTCCCTGAGACTCTCCTG | **CTTA**ACCTGAGGAGACGGTGACC |

Libraries were prepared by using the IgH-VH3 family rearrangement (previously identified to be rearranged by all samples included in this study).

In total 38 amplicon libraries were prepared: 24 from flow-sorted sub-populations, 13 from NS samples and 1 control library (not shown) prepared using the melanoma cell line A375 and Ig germline primers (Campbell, PNAS 2009). Two libraries included in the 454 sequencing were prepared using CD77 in the flow-sorting preparation as discriminator marker for CB and CC. In this table only patient’s samples and the sequences of the multiplex identifier (MID) tag IgH-VH3/JH primers are shown; used for the preparation of the are not shown.

Highlighted in grey are shown the MID tags specific for each library.

In bold are shown the 2 libraries that did not work on the 454 Sequencer (R0012NS and R0012 CC CD77-) and the one that was excluded by further analysis (R1381 CC CD77-).

^ Samples obtained after flow-sorting by using CD77 as a marker for CB.

± Sample R1381 not sorted was used to prepare 2 different libraries, using 2 primers having 2 different bar-codes.

* Sample R8403 not sorted was used for preparing 6 different libraries using the same set of primers.
