# Supplementary material for: High Throughput Sequencing Analysis of the Immunoglobulin Heavy Chain Gene from Flow-Sorted B Cell Sub-Populations Define the Dynamics of Follicular Lymphoma Clonal Evolution
Source: PLoS One. 2015 Sep 1;10(9):e0134833. doi: 10.1371/journal.pone.0134833 (PMC4556522; doi:10.1371/journal.pone.0134833)
Supplement: S3 Table — (DOC) [file pone.0134833.s010.doc]

**S3 Table Number and % of clones detected in the NS and sorted populations**

| **Patient** | **Sample** | **Library** | **Total No clones (not shared)±** | **%Total Clones** | **%Not shared Clones** |
| --- | --- | --- | --- | --- | --- |
| 1* | R0012 - tFL | PGC | 37 (35) | 18 | 95 |
|  |  | CB | 60 (46) | 28 | 77 |
|  |  | CC | 84 (68) | 40 | 81 |
|  |  | ME | 30 (18) | 14 | 60 |
|  |  |  |  |  |  |
|  | R1381 - FL1 | NS | 8 (3) | 6 | 38 |
|  |  | PGC | 70 (45) | 51 | 64 |
|  |  | ME | 60 (28) | 43 | 47 |
|  |  |  |  |  |  |
|  | R2005 - FL2 | NS | 60 (40) | 15 | 67 |
|  |  | PGC | 70 (64) | 17 | 91 |
|  |  | CB | 106 (59) | 26 | 56 |
|  |  | CC | 116 (80) | 27 | 69 |
|  |  | ME | 63 (33) | 15 | 52 |
|  |  |  |  |  |  |
| 2 | R1655 - FL1 | NS | 30 (22) | 25 | 73 |
|  |  | CB | 68 (57) | 56 | 84 |
|  |  | CC | 23 (21) | 19 | 91 |
|  |  |  |  |  |  |
|  | R3878 - FL2 | NS | 101 (73) | 26 | 72 |
|  |  | CB | 151 (122) | 40 | 79 |
|  |  | CC | 130 (100) | 34 | 77 |
|  |  |  |  |  |  |
| 3 | R8403 - FL1 | NS | 76 (32) | 24 | 42 |
|  |  | PGC | 76 (44) | 24 | 58 |
|  |  | CB | 75 (41) | 23 | 55 |
|  |  | CC | 88 (43) | 27 | 49 |
|  |  | ME | 7 (3) | 2 | 43 |
|  |  |  |  |  |  |
|  | R9129 - FL2 | NS | 145 (79) | 22 | 54 |
|  |  | PGC | 69 (26) | 11 | 38 |
|  |  | CB | 188 (86) | 29 | 46 |
|  |  | CC | 221 (119) | 34 | 54 |
|  |  | ME | 23 (8) | 4 | 34 |
|  |  |  |  |  |  |

* The R1381 CC CD77- population, not included in the final analysis, and the R0012NS population that did not pass the 454 sequencing filtering process are not shown.

± Between brackets are indicated the number of unique clones (not shared with any other library) detected in each library. In total we identified 519 unique clones in pt1, 395 in pt2 and 481in pt3.
