# Supplementary material for: High Throughput Sequencing Analysis of the Immunoglobulin Heavy Chain Gene from Flow-Sorted B Cell Sub-Populations Define the Dynamics of Follicular Lymphoma Clonal Evolution
Source: PLoS One. 2015 Sep 1;10(9):e0134833. doi: 10.1371/journal.pone.0134833 (PMC4556522; doi:10.1371/journal.pone.0134833)
Supplement: S4 Table — (DOC) [file pone.0134833.s011.doc]

**S4 Table Characteristics of the PGC and ME sub-populations**

| **Pt** | **Sample** | **Sub-population** | **No (%) Clones homologous GL*** | **No or range SHM different from MC±** | **No (%) Clones with SHM in DH-JH #** |
| --- | --- | --- | --- | --- | --- |
| 1 | R0012- tFL | PGC | 36 (97) | 1 | 24 (65) |
|  |  | CB | 0 (0) | 1-4 | 25 (42) |
|  |  | CC | 1 (1) | 3 | 18 (21) |
|  |  | ME | 3 (10) | 2 | 15 (50) |
|  |  |  |  |  |  |
|  | R1381 - FL1 | PGC | 3 (4) | 1-4 | 25 (36) |
|  |  | ME | 1 (2) | 1-4 | 21 (35) |
|  |  |  |  |  |  |
|  | R2005 - FL2 | PGC | 68 (97) | 1-3 | 20 (28) |
|  |  | CB | 2 (2) | 1-5 | 19 (18) |
|  |  | CC | 1 (1) | 1-6 | 28 (24) |
|  |  | ME | 2 (3) | 2 | 48 (76) |
|  |  |  |  |  |  |
| 2 | R1655-FL1 | CB | 0 (0) | 1-5 | 13 (19) |
|  |  | CC | 2 (9) | 1-4 | 2 (9) |
|  |  |  |  |  |  |
|  | R3878-FL2 | CB | 0 (0) | 1-4 | 77 (51) |
|  |  | CC | 0 (0) | 1-6 | 76 (58) |
|  |  |  |  |  |  |
| 3 | R8403 - FL1 | PGC | 1 (1) | 1-8 | 7 (9) |
|  |  | CB | 0 (0) | 1-8 | 5 (7) |
|  |  | CC | 0 (0) | 1-9 | 10 (11) |
|  |  | ME | 4 (57) | 2 | 4 (57) |
|  |  |  |  |  |  |
|  | R9129 - FL2 | PGC | 0 (0) | 1-4 | 3 (4) |
|  |  | CB | 0 (0) | 1-4 | 6 (3) |
|  |  | CC | 0 (0) | 1-5 | 8 (4) |
|  |  | ME | 0 (0) | 1-3 | 0 (0) |
|  |  |  |  |  |  |

* No of clones with a VH >90% homologous to the germline sequence

± Clones homologous to the germline sequence have been excluded from this analysis

# Clones with at least 1 SHM mapping in the DH-JH region.
