# Supplementary material for: High Throughput Sequencing Analysis of the Immunoglobulin Heavy Chain Gene from Flow-Sorted B Cell Sub-Populations Define the Dynamics of Follicular Lymphoma Clonal Evolution
Source: PLoS One. 2015 Sep 1;10(9):e0134833. doi: 10.1371/journal.pone.0134833 (PMC4556522; doi:10.1371/journal.pone.0134833)
Supplement: S5 Table — (DOC) [file pone.0134833.s012.doc]

**Table S5 Prevalence of the haplotypes shared among the Pt1 samples and Pt2 samples**

| **Patient** | **Sub-clones** | **Prevalence R0012-tFL** | **Prevalence R1381 FL1** | **Prevalence**  **R2005 FL2** |
| --- | --- | --- | --- | --- |
| 1 | a116t | 0.0008 | 0.0011 | 0.0021 |
|  | a134c | 0.0013 | 0.0010 | 0.0007 |
|  | a244t | 0.0038 | 0.0010 | 0.0010 |
|  | a249t | 0.0013 | 0.0008 | 0.0008 |
|  | a53t | 0.0016 | **0.0036** | **0.009** |
|  | *c33t.g266c* | **0.0009** | **0.0011** | 0.0001 |
|  | c48g | 0.0025 | **0.0212** | **0.0014** |
|  | c48g.c49t | **0.0055** | **0.0007** | 0.0001 |
|  | c54g | **0.0234** | **0.0372** | **0.0069** |
|  | *c54g.g266c* | **0.0006** | **0.0018** | **0.0013** |
|  | c70g | 0.0021 | **0.0026** | 0.0062 |
|  | g180c | 0.0020 | 0.0017 | 00017 |
|  | *g266c* | **0.1056** | **0.0219** | **0.0423** |
|  | g63a | 0.281 | **0.1611** | **0.0853** |
|  | g63a.a208g | 0.0003 | 0.0003 | 0.0009 |
|  | g63c | **0.0101** | **0.0102** | **0.0457** |
|  | g63t | 0.0018 | **0.0071** | **0.0028** |
| **Patient** | **Sub-clones** | **Prevalence R1655** | **Prevalence R3878** |  |
| 2 | c32a | 0.0006 | 0.0020 |  |
|  | g125c | 0.0012 | **0.0688** |  |
|  | g219c | 0.0012 | 0.0027 |  |
|  | g31c | 0.0007 | 0.0023 |  |
|  | g31t | 0.0031 | **0.0041** |  |

In bold are shown those haplotypes detected in more than 1 sub-population.

In Italic are shown the3 shared sub-clones that carry SHM outside the VH region and therefore excluded from the Lineage Tree Analysis.

The prevalence shown is calculated as total prevalence.
